# Chinmedomics strategy for elucidating the effects and effective constituents of Danggui Buxue Decoction in treating blood deficiency syndrome

**DOI:** 10.3389/fmolb.2024.1376345

**Published:** 2024-03-15

**Authors:** Ye Zhang, Yu Yang, Junling Ren, Guangli Yan, Le Yang, Xiuhong Wu, Ling Kong, Hui Sun, Ying Han, Xiwu Zhang, Xijun Wang

**Affiliations:** ^1^ State Key Laboratory of Integration and Innovation of Classic Formula and Modern Chinese Medicine, National Chinmedomics Research Center, National TCM Key Laboratory of Serum Pharmacochemistry, Metabolomics Laboratory, Department of Pharmaceutical Analysis, Heilongjiang University of Chinese Medicine, Harbin, China; ^2^ State Key Laboratory of Quality Research in Chinese Medicine, Macau University of Science and Technology, Macao, China; ^3^ State Key Laboratory of Dampness Syndrome, The Second Affiliated Hospital Guangzhou University of Chinese Medicine, Guangzhou, China

**Keywords:** Danggui Buxue Decoction, blood deficiency syndrome, metabolomics, chinmedomics, effective constituents, quality markers

## Abstract

**Introduction:**

Danggui Buxue Decoction (DBD) is a clinically proven, effective, classical traditional Chinese medicine (TCM) formula for treating blood deficiency syndrome (BDS). However, its effects and effective constituents in the treatment of BDS remain unclear, limiting precise clinical therapy and quality control. This study aimed to accurately evaluate the effects of DBD and identify its effective constituents and quality markers.

**Methods:**

BDS was induced in rats by a combined injection of acetylphenylhydrazine and cyclophosphamide, and the efficacy of DBD against BDS was evaluated based on body weight, body temperature, energy metabolism, general status, visceral indices, histopathology, biochemical markers, and metabolomics. The effects of DBD on urinary and serum biomarkers of BDS were investigated, and the associated metabolic pathways were analyzed via metabolomics. Guided by Chinmedomics, the effective constituents and quality markers of DBD were identified by analyzing the dynamic links between metabolic biomarkers and effective constituents *in vivo*.

**Results:**

DBD improved energy metabolism, restored peripheral blood and serum biochemical indices, and meliorated tissue damage in rats with BDS. Correlation analyses between biochemical indices and biomarkers showed that 15(S)-HPETE, LTB4, and taurine were core biomakers and that arachidonic acid, taurine, and hypotaurine metabolism were core metabolic pathways regulated by DBD. Calycosin-7-glucoside, coumarin, ferulic acid sulfate, cycloastragenol, (Z)-ligustilide + O, astragaloside IV, acetylastragaloside I, and linoleic acid were identified as effective constituents improving the hematopoietic function of the rats in the BDS model. Additionally, calycosin-7-glucoside, ferulic acid, ligustilide, and astragaloside IV were identified as quality markers of DBD.

**Conclusion:**

The hematopoietic function of DBD was confirmed through analysis of energy metabolism, biochemical markers, histopathology, and metabolomics. Moreover, by elucidating effective constituents of DBD in BDS treatment, quality markers were confirmed using a Chinmedomics strategy. These results strengthen the quality management of DBD and will facilitate drug innovation.

## 1 Introduction

Blood deficiency syndrome (BDS) is a fundamental syndrome in traditional Chinese medicine (TCM) and is caused by insufficient blood and excessive consumption (Sun et al., 2022). The incidence of BDS is predicted to increase dramatically, leading to detrimental health effects including headaches, weakened immune systems, and infertility (Huang et al., 2020; Wang et al., 2021; Gong et al., 2023). Although blood transfusion is the current clinical therapy, it may lead to allergic reactions and gastrointestinal discomfort, and BDS may recur upon discontinuation of treatment (Kurihara et al., 2016). Given the complexity of BDS pathology and its limited therapeutic options, exploration of alternative therapies is vital.

Danggui Buxue Decoction (DBD), a classical TCM prescription for BDS, is first mentioned in “Neiwaishang Bianhuo Lun” by Li Dongyuan in the Jin Dynasty. DBD consists of dried *Astragali radix* (dried root of *Astragalus mongholicus* Bunge) and *Angelica sinensis* (dried root of *Angelica sinensis* Diels) in a mass ratio of 5:1, and is commonly used for its blood-tonifying effects (Hua et al., 2019; Tie et al., 2022). DBD has shown promising therapeutic outcomes in patients with focal cerebral ischemia, ischemic heart disease, and diabetic atherosclerosis (Zhang et al., 2006; Dong et al., 2022; Dou et al., 2023), and particularly in treating BDS (Shi et al., 2014). DBD contains abundant chemical constituents, notably, organic acids, saponins, volatile oils, and flavonoids, which may be active in BDS treatment (Shi et al., 2014; Liu et al., 2019; Chen T. et al., 2022). However, the mechanism and exact *in vivo* constituents mediating the effects of DBD in BDS treatment remain unconfirmed, hindering clinical applications and pharmaceutical innovations. Therefore, we aimed to determine the constituents associated with DBD treatment of BDS *in vivo* using the Chinmedomics strategy.

**FIGURE 1:**
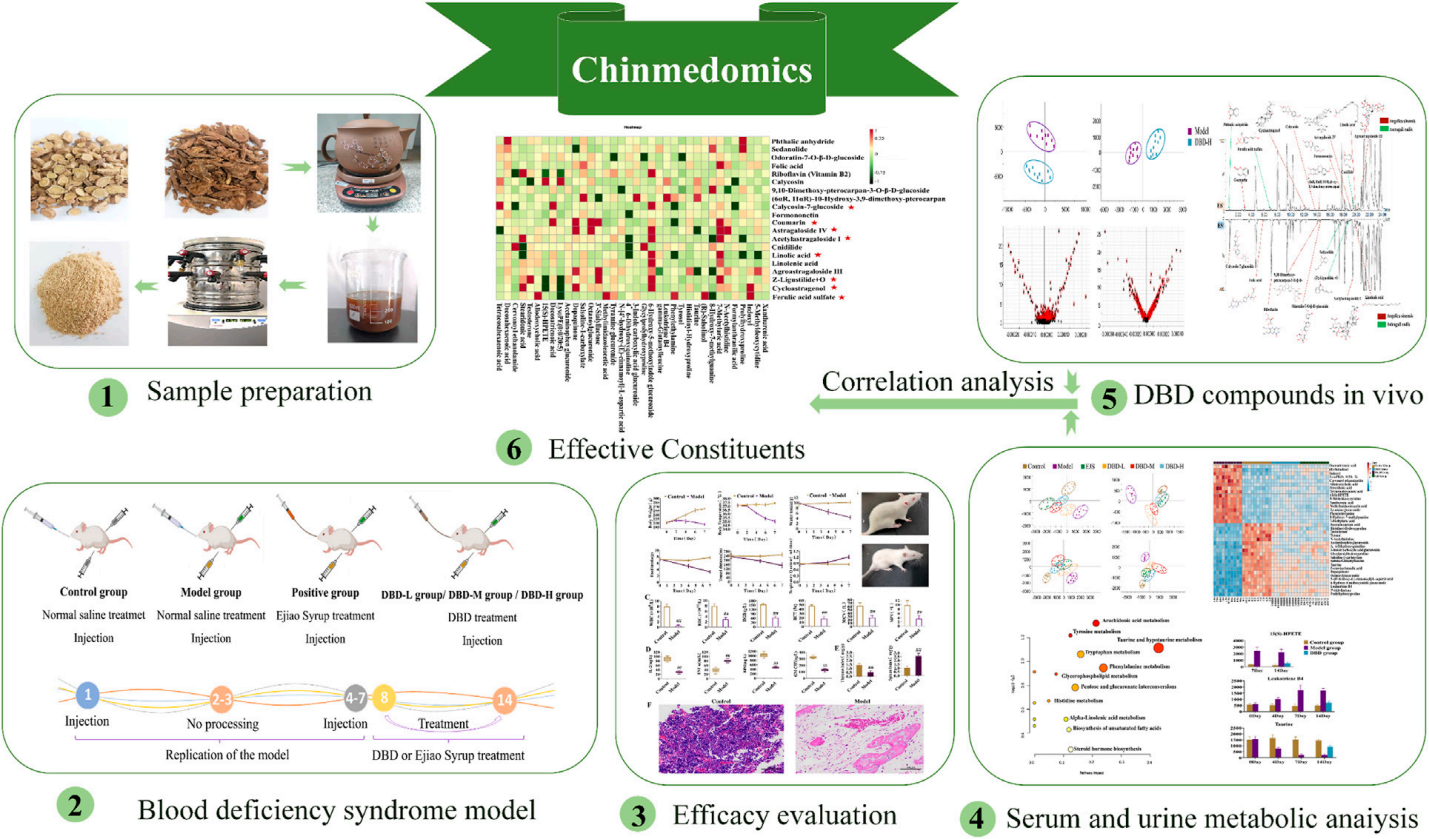
Research process for effective compounds of DBD based on chinmedomics strategy.

Chinmedomics is a powerful strategy for evaluating and characterizing the effects of TCM-based treatments (Sun et al., 2019; Wang et al., 2019; Xiong et al., 2020). It enables the identification of effective constituents and the mechanisms underlying efficacy (Han et al., 2020; Ren et al., 2020). Additionally, this strategy addresses the challenge of multi-component TCM treatments functioning on multiple targets, allowing the exploration of diverse biological systems under varying conditions (Han et al., 2020). Hence, in this study, we employed a Chinmedomics approach to investigate the efficacy of DBD in BDS, screened *in vivo* DBD constituents based on effectiveness, and identified potential effective constituents by analyzing the kinetic relationships between biomarkers and components *in vivo*, thereby identifying DBD quality markers (Figure 1).

## 2 Materials and methods

### 2.1 Reagents and drugs

*Astragali radix* (lot number: JL001-180610) and *Angelicae sinensis radix* (lot number: JL212-180610) were donated by Harbin Traditional Chinese Medicine Fourth Factory Co., Ltd. (Harbin, China). Ejiao Syrup (EJS, lot number: 2212029) was provided by Dong-E-E-Jiao Co., Ltd. (Shandong, China). Cyclophosphamide was procured from Jiangsu Hengrui Pharmaceutical Co., Ltd. (approval number: 22111425; Jiangsu, China). Injectable acetylphenylhydrazine was donated by Shanghai Yuanye Bio-Technology Co., Ltd. (lot number: M17HS178436; Shanghai, China). HPLC functional acetonitrile and methanol were supplied by Thermo Fisher Scientific (Waltham, MA, United States). Sodium chloride was obtained from Harbin Sanlian Pharmaceutical Co., Ltd. (Harbin, China). Distilled water was provided by Watsons Co., Ltd. (Guangzhou, China). ELISA kits for detecting rat serum interleukin-2 (IL-2), tumor necrosis factor-α (TNF-α), erythropoietin (EPO), and macrophage colony-stimulating factor (GM-CSF) were purchased from Nanjing Jiancheng Institute of Biotechnology Co., Ltd. (Nanjing, China).

### 2.2 Preparation of DBD

The decoction method mentioned in the classical book “Neiwaishang Bianhuo Lun” was used to prepare the DBD samples. *Astragali radix* (41.3 g) and *Angelicae sinensis radix* (8.26 g) were soaked in 600 mL of water for 40 min and then decocted until the water level reached 300 mL. A 160-mesh filter cloth was used to filter the decoction, which was freeze-dried for 12 h at ˗50°C to acquire a yellow powder. The average yield of the powder was 15.57% (n = 15). The levels of ferulic acid, calycosin-7-glucoside, and astragaloside IV were measured and recorded as indicators of the quality of lyophilized DBD powder. In the five batches of powder, the corresponding relative standard deviations for the aforementioned compounds were 0.35%, 0.59%, and 1.74% (Supplementary Figure S1; Supplementary Table S1). Fingerprints from the 15 batches of DBD had similarity scores exceeding 0.93 (Supplementary Figure S2; Supplementary Table S2), signifying the stability and controllability of the samples.

### 2.3 Animals model

**FIGURE 2:**
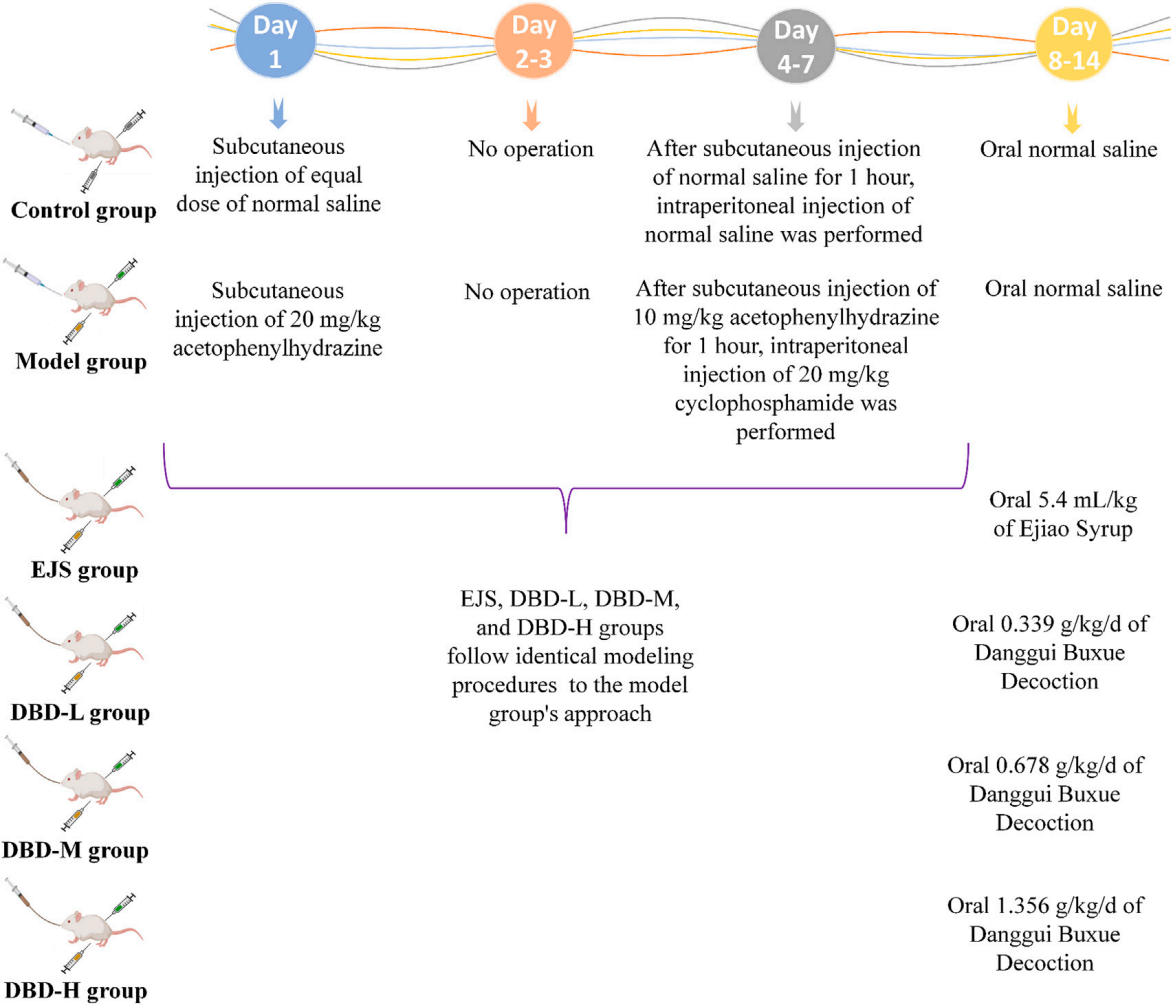
Preparation and treatment of the BDS animal model generated by Med Peer (www.medpeer.cn).

Eighty male Sprague-Dawley rats (body weight: 200 ± 15 g) were purchased from Liaoning Changsheng Biotechnology Co., Ltd. (license no.: SCXK (Liao) 2020-0001; Benxi, China). The rats were housed in a controlled environment with a temperature range of 22°C–25°C, an average humidity level of 45%–55%, a 12 h light-dark cycle, and unlimited access to food and water. After a week of acclimatization, the rats were allocated into 8 experimental groups: control group 1 (Control 1; n = 10), control group 2 (Control 2; n = 10), model group 1 (Model 1; n = 10), model group 2 (Model 2; n = 10), Ejiao Syrup group (EJS; n = 10), low-dose DBD group (DBD-L; n = 10), medium-dose DBD group (DBD-M; n = 10), and high-dose DBD group (DBD-H; n = 10). Rats in the model and treatment groups were administered hypodermic injections of 2% acetylphenylhydrazine at varying doses on days 1 (20 mg/kg) and 4 (10 mg/kg), followed by an intraperitoneal injection of cyclophosphamide (20 mg/kg) 1 h after subcutaneous injection of 2% acetophenazine (10 mg/kg) from days 4–7 to replicate the BDS model; control rats received an equivalent dose of injected saline. On day 8, all Control 1 and Model 1 rats were sacrificed for BDS model evaluation. Lyophilized DBD powder and EJS solutions were administered orally to the respective treatment groups at different doses on days 8–14. The DBD-L group received half of the human clinical dosage (0.339 g/kg/d), the DBD-M group an equal dose (0.678 g/kg/d), the DBD-H group twice the dose (1.356 g/kg/d), and the EJS group received continuous EJS by gavage at an equivalent human dose (5.4 mL/kg). On the 15th day, all rats were sacrificed to evaluate treatment efficacy. Figure 2 illustrates the animal modeling process and mode of drug administration. The Ethics Committee of Heilongjiang University of Chinese Medicine approved the study protocol (2023062507), and all investigations were conducted in compliance with the Declaration of Helsinki.

### 2.4 Evaluation of the BDS model and DBD efficacy

#### 2.4.1 Energy metabolism

We measured water and food intake, travel distance, and respiratory quotient as indices of energy metabolism in the rats to evaluate the BDS model and the effectiveness of DBD. The indices were measured using a Promethion Metabolic System (Sable Systems, North Las Vegas, NE, United States) on the 4th, 7th, 11th, and 14th days of the experiment.

#### 2.4.2 Peripheral blood test

A portion of the blood samples collected on days 8 and 15 were rapidly agitated in anticoagulant tubes to prevent coagulation and to measure the hematocrit (HCT), red blood cells (RBC), hemoglobin (HGB), white blood cells (WBC), mean platelet volume (MPV), and mean corpuscular volume (MCV) in rat blood using an automatic blood cell analyzer (XE-5000; Kobe, Japan).

#### 2.4.3 Biochemical analysis

The blood was kept for 1 h to permit serum separation from the blood cells, and then centrifuged at 3,500 rpm for 10 min at 4°C to extract the serum. The IL-2, TNF-α, EPO, and GM-CSF content in the serum was determined using an automatic biochemical analyzer (FlexA-200; Hangzhou, China) according to the manufacturer’s instructions.

#### 2.4.4 Histopathology analysis

After blood collection, the thymus, spleen, and femurs of the rats were isolated, cleaned with physiological saline solution, and desiccated using filter paper. The weights of each spleen and thymus were documented to calculate visceral indices (visceral index = visceral mass (mg)/body mass (g)). Fresh femurs were fixed in 4% paraformaldehyde for 48 h. Afterwards, portions of the tissue were stained with hematoxylin and eosin (H&E) and examined at 200× magnification using a light microscope (Eclipse Ci-L, Nikon, Tokyo, Japan).

### 2.5 Metabolomics analysis

#### 2.5.1 Sample gathering and preparation

##### 2.5.1.1 Urine samples

Urine samples were gathered over a 12 h period (7:30 p.m. to 7:30 a.m.) and subsequently centrifuged at 13,000 rpm for 10 min at 4°C on the 0th, 4th, 7th, and 14th days of the experiment. The supernatant was subjected to two-fold dilution using ultrapure water, followed by vortex mixing for 1 min, centrifugation at 13,000 rpm for 10 min at 4°C, and 0.22 μm filtration for subsequent ultra-high-performance liquid chromatography with quadrupole time-of-flight mass spectrometry (UPLC-Q/TOF-MS) analysis.

##### 2.5.1.2 Serum samples

Serum samples were collected from the Control 1 and Model 1 groups on day 8 of the study, whereas samples from the remaining 6 groups were obtained after 7 days of therapy. All specimens were gathered from the gastrointestinal aorta, and the serum and blood cells were allowed to separate for 1 h. The samples were then centrifuged at 3,500 rpm for 10 min at 4°C to gather the serum. The supernatant was mixed with three times its volume of methanol and vortexed for 1 min before being centrifuged at 13,000 rpm for 10 min at 4°C. Afterwards, 600 μL of serum supernatant was dried with nitrogen at 38°C, resuspended with 200 μL of methanol, and further centrifuged for 10 min under the same conditions as before. Finally, the supernatant was passed through a 0.22 μm filter prior to ultra-high-performance liquid chromatography-mass spectrometry (UPLC-MS) analysis.

#### 2.5.2 LC–MS analysis

##### 2.5.2.1 Urinary metabolomics analysis

Chromatographic analysis was performed using a Waters Acquity™ UPLC system (Waters Corporation, Milford, MA, United States). An Acquity™ UPLC HSS T_3_ column (1.8 μm, 2.1 × 100 mm; Waters) was used to separate the urine samples at 35°C. The amount of fluid injected was 4 μL, and the optimal velocity of flow was 0.3 mL/min. Phase A comprised 0.1% (v/v) formic acid mixed with acetonitrile, and phase B comprised 0.1% (v/v) formic acid in water. The following were the parameters for the elution of the urine: 0–2.5 min, 1%–5% A; 2.5–5 min, 5%–8% A; 5–7 min, 8%–11% A; 7–8 min, 11%–22% A; 8–11 min, 22%–35% A; 11–12 min, 35%–99% A. Mass spectroscopy measurement was accomplished on a Synapt™ G2-Si MS system (Waters) equipped with electrospray ionization (ESI). The atomizing and cone gases used in the analysis were both nitrogen, with flow rates of 50 and 700 L/h, respectively, at a temperature of 380°C; the operating temperature of the ion source was 110°C. The urine data were collected using capillary electrical voltages of 2.7 kV (ESI^+^) and 2.3 kV (ESI^−)^, along with a cone voltage of 40 V. The information-gathering rate was set at 0.2 s/scan, and the full scan range was m/z 50–1,200 Da.

##### 2.5.2.2 Serum metabolomics analysis

The analytical system, column, and flow rate used for serum chromatography were identical to those used for the urine analysis. The amount of fluid injected was 2 μL, and the ambient temperature in the column was 35°C. The detailed serum linear gradients were as follows: 0–3 min, 1%–15% A; 3–5 min, 15%–29% A; 5–7 min, 29%–62% A; 7–8 min, 62%–65% A; 8–11 min, 65%–68% A; 11–12 min, 68%–99% A. Serum mass spectrometry conditions were consistent with those in urine analysis.

#### 2.5.3 Multivariate analysis and identification of potential biomarkers

Unprocessed UPLC-MS data were uploaded into Progenesis QI software (version 2.0; Waters) for noise elimination, maximum acquisition, alignment, selection, and standardization to obtain the ion retention time-*m/z* ratio-peak comparative intensity matrix. Normalized results were then transferred into EZinfo 3.0 (Waters) for the analysis of multiple variables. Principal component analysis (PCA) was used to identify general variations between groups, whereas orthogonal partial least squares discriminant analysis (OPLS-DA) was employed to discern the characteristic compounds that varied within groups and to validate the BDS model. On the basis of fold change (FC) > 1.5, t-tests of intergroup changes (*p* < 0.05), and variable projection importance (VIP) > 1, potential BDS biomarkers in urine and serum were identified.

To determine the Rt-*m/z* of potential biomarkers, relative standards and Internet databases such as HMDB (https://hmdb.ca) were employed. The MS/MS data were then entered via the MassLynx-nested MassFragment™ application manager (Waters) for structural verification and to align fragment masses. To further elucidate the metabolic pathways involved, BDS-related differential metabolites were screened using MetaboAnalyst 5.0 (http://www.metaboanalyst.ca/).

### 2.6 Analysis of the effective constituents of DBD

#### 2.6.1 Sample preparation

A total of 0.2 g of freeze-dried DBD powder was precisely weighed and mixed with 2 mL of methanol. The solution was extracted ultrasonically for 45 min and centrifuged at 13,000 rpm for 10 min at 4°C, and the resulting liquid was passed through a 0.22 μm filter prior to UPLC-MS analysis. Forty microliters of phosphoric acid were transferred to 2 mL of serum. The resulting mixture was packed onto a preactivated Oasis hydrophilic-lipophilic balanced (HLB) C18 extraction column with a solid solvent (Waters) for *in vivo* enrichment analysis of DBD constituents. The column was then eluted with 2 mL of methanol, and the filtrate was separated and dehydrated with nitrogen at 38°C. Finally, the substance was totally dispersed in 150 μL of 60% methanol, followed by centrifugation at 13,000 rpm for 15 min at 4°C.

#### 2.6.2 Analysis condition

Chromatographic analysis was conducted employing an Acquity™ UPLC system (Waters, Milford, United States) coupled with an HSS T3 column (1.8 μm, 2.1 × 100 mm; Waters) and using mobile phases A (0.1% v/v formic acid mixed with acetonitrile) and B (0.1% v/v formic acid mixed with water). The gradient profile was as follows: 0–2 min, 1%–6% A; 2–5 min, 6%–10% A; 5–6 min, 10%–15% A; 6–10 min, 18%–20% A; 10–11 min, 20%–23% A; 11–16 min, 23%–29% A; 16–18 min, 29%–50% A; 18–21 min, 50%–60% A; 21–24 min, 60%–100% A. In addition, the average flow speed was 0.4 mL/min, the ambient temperature of the column was 35°C, and the amount of fluid infused was 4 μL.

Mass spectrometry analysis was conducted using a Waters Synapt™ G2-Si MS^E^ mass spectrometer (Waters, United States) fitted with ESI. Expect for the operating mode, the mass spectrometry conditions were identical to those employed for the serum and urine samples described in Section 2.5.2.

#### 2.6.3 Constituent analysis and identification

All data were entered into Progenensis QI to obtain ion retention time-*m/z*-peak comparative intensity and then into Ezinfo for PCA and OPLS-DA, in conjunction with the acquired VIP, to identify divergent ions formed between two sets of data. The metabolites of the parent constituents were identified using the PubChem and ChemSpider databases and the MassFragment and MetaboAnalyst programs, coupled with a literature review. Plausible chemical formulae, accurate masses, and MS/MS fragmentation of ions further confirmed the presence of DBD elements *in vivo* and *in vitro*.

### 2.7 Analysis of the relationship between absorbed constituents and biomarkers

The correlation coefficients of the biomarkers in the BDS model and the effective constituents *in vivo* were determined using the Pearson correlation analysis platform. By monitoring the link between the marker substrates and plasma PCMS chemicals, the correlation coefficient (r) was computed to identify potential effective constituents. In this study, compounds with significant correlation coefficients (r = 0.75) were identified, and those with 6 or more closely linked biomarkers were deemed to be effective constituents of DBD for the treatment of BDS in rats.

## 3 Results

### 3.1 Evaluation of the BDS model

**FIGURE 3:**
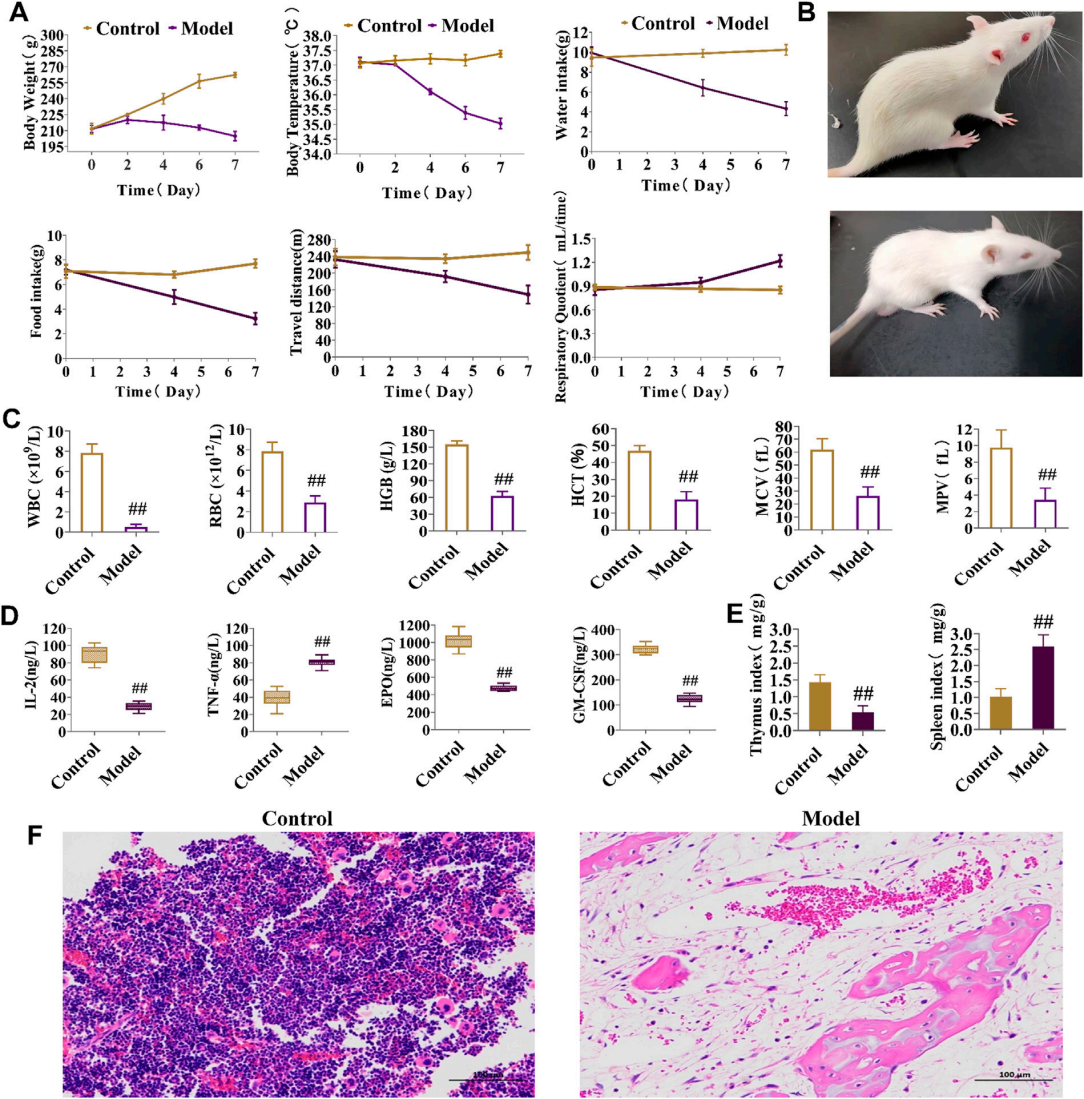
Behavioral, energy metabolism, biochemical, and pathological analysis for the assessment of BDS. **(A)** Body weight, body temperature, water intake, food intake, travel distance, and respiratory quotient in energy metabolism detection. **(B)** General status of the control group (up) and model group (down). **(C)** The level of HGB, RBC, WBC, HCT, MPV, and MCV in peripheral blood. **(D)** The level of IL-2, TNF-α, EPO, and GM-CSF in rat serum. **(E)** The thymus index and spleen index. **(F)** Results of the histopathology analysis of the rat marrow stained with eosin and hematoxylin (×200). ^#^ *p* < 0.05, ^##^ *p* < 0.01 vs. control group.

Weight and body temperature decreased during the modeling process in the experimental group compared to those in the control group, with significantly lower water intake, food intake, and travel time (measures of energy metabolism), whereas the respiratory quotient was noticeably greater (Figure 3A). By the final day of BDS model formation, in contrast to the control group, the eyes, ears, and paws of the developing model rats were bloodless (Figure 3B), consistent with the clinical symptoms. Peripheral blood levels of HCT, MPV, WBC, RBC, HGB, and MCV were considerably lower than those in the control group (Figure 3C). According to biochemical analysis, the serum content of TNF-α substantially increased in the model group, whereas those of IL-2, EPO, and GM-CSF dramatically declined when compared to values in the control group (Figure 3D). Figure 3E demonstrates that, in comparison to the control group, the BDS group was characterized by a decrease in the thymus index and an increase in the spleen index. H&E staining (Figure 3F) revealed bone marrow destruction, adipose tissue growth, and a large reduction in the number of hematopoietic cells. These indicators collectively indicated abnormalities in both the hematopoietic and immunological functions of the rats in the model group.

### 3.2 Metabolomics analysis of rats in the BDS model

**FIGURE 4:**
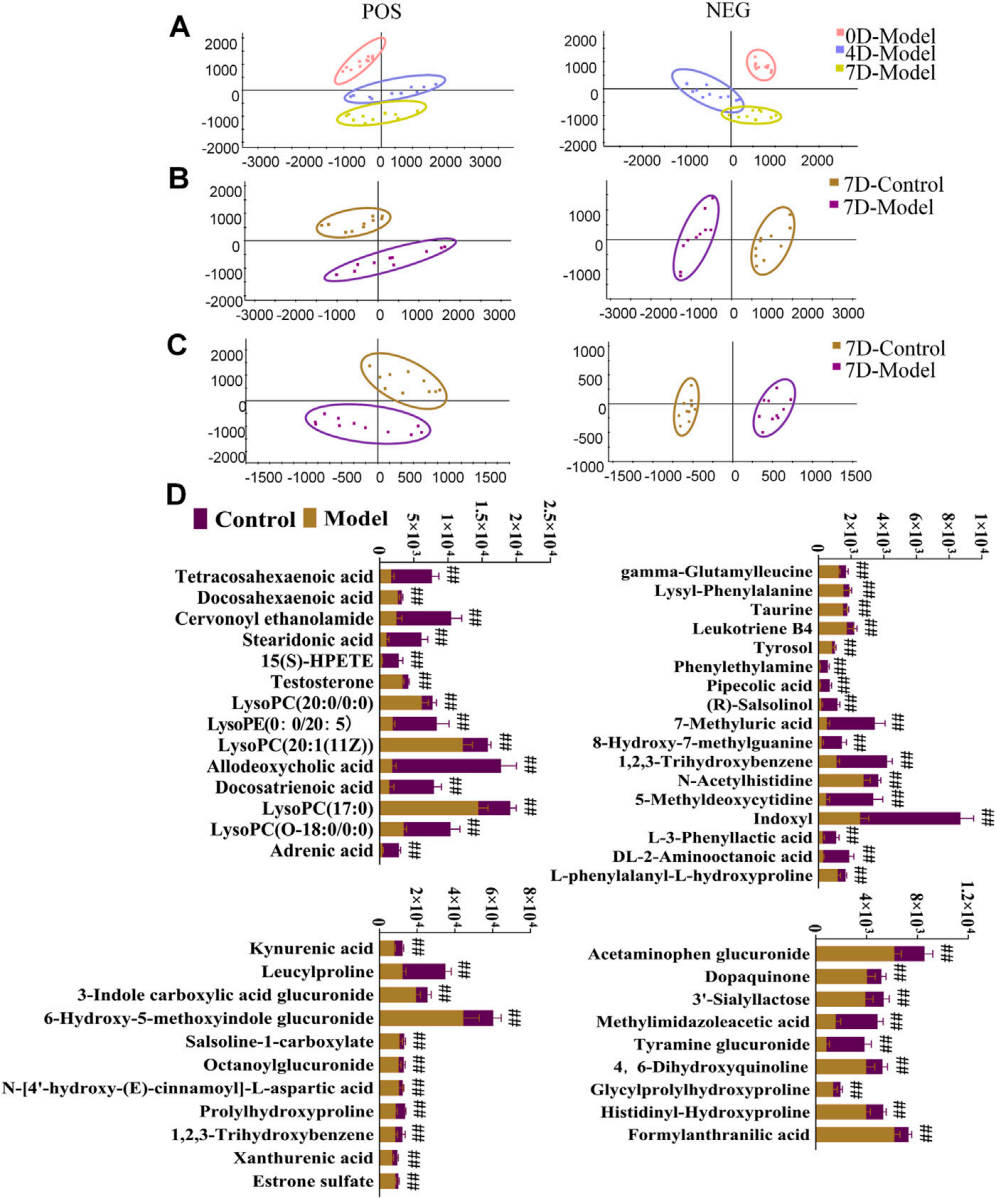
Urine and serum sample multivariate analysis comparing the model and control groups. **(A)** PCA scores plot of rat urinary metabolism at various time points in modeling blood deficiency syndrome; **(B)** PCAs illustrate distinct urine profiles for control and model groups; **(C)** PCAs illustrate distinct serum profiles for control and model groups. **(D)** Relative contents for potential biomarkers in urine and serum for the model and control groups. ^#^ *p* < 0.05, ^##^ *p* < 0.01 vs. control group.

Serum and urine samples were analyzed, and diagrams comparing the control and model groups are shown in Supplementary Figures S3, S4. PCA score plots for rat urine metabolism data indicated that the metabolic trajectory of the model group gradually shifted away from that of the control group during BDS replication on days 4 and 7 (Figure 4A). Serum and urine metabolic data were evaluated using PCA and OPLS-DA on day 7 of modeling. Notably, the serum and urine metabolic profiles of rats in the model group showed considerable changes in metabolic circuit (Figure 4B, C). The control and model groups were screened for 14 serum and 39 urine biomarkers possibly related to BDS, characterized by *p* < 0.05, fold-change >1.5, and VIP >1 (Supplementary Table S3). The variance in BDS biomarker levels between the control and model groups is visually displayed using a stacked bar plot (Figure 4D).

### 3.3 Analysis of metabolic pathways associated with BDS

**FIGURE 5:**
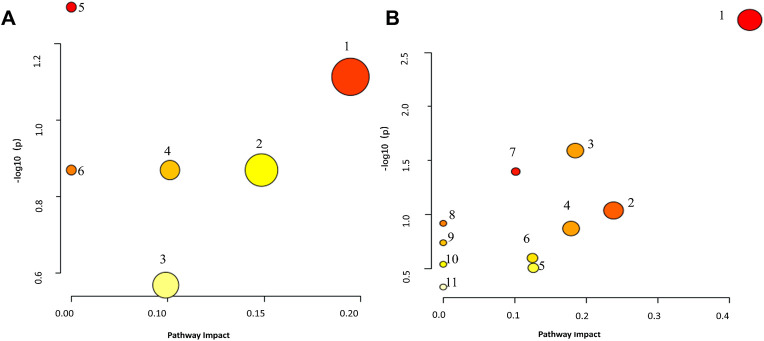
Metaboanalyst Pathway Analysis elucidates the disorderly pattern of serum/urine metabolite pathways. **(A)** serum metabolites relative pathways, 1: Arachidonic acid metabolism, 2: Biosynthesis of unsaturated fatty acids, 3: Steroid hormone biosynthesis, 4: Alpha-Linolenic acid metabolism, 5: Glycerophospholipid metabolism, 6: Ether lipid metabolism. **(B)**: urine metabolites relative pathways, 1: Taurine and hypotaurine metabolism, 2: Arachidonic acid metabolism, 3: Steroid hormone biosynthesis, 4: Pentose and glucuronate interconversions, 5: Tryptophan metabolism, 6: Phenylalanine metabolism, 7: Tyrosine metabolism, 8: Caffeine metabolism, 9: Histidine metabolism, 10: Lysine degradation, 11: Primary bile acid biosynthesis.

To investigate the metabolic pathways affected by BDS in the rat model, 14 serum and 39 urinary biomarkers were identified and entered into MetaboAnalyst 5.0. Four metabolic pathways in serum and seven in urine were found to be most substantially linked with BDS when an influence >0.1 was used as the evaluation requirement. The metabolism of arachidonic acid in serum and taurine and hypotaurine in urine displayed the biggest departure from the coordinate axis and the deepest red color, as depicted in Figure 5 and Supplementary Tables S4, S5.

### 3.4 Therapeutic effects of DBD on BDS in rats

**FIGURE 6:**
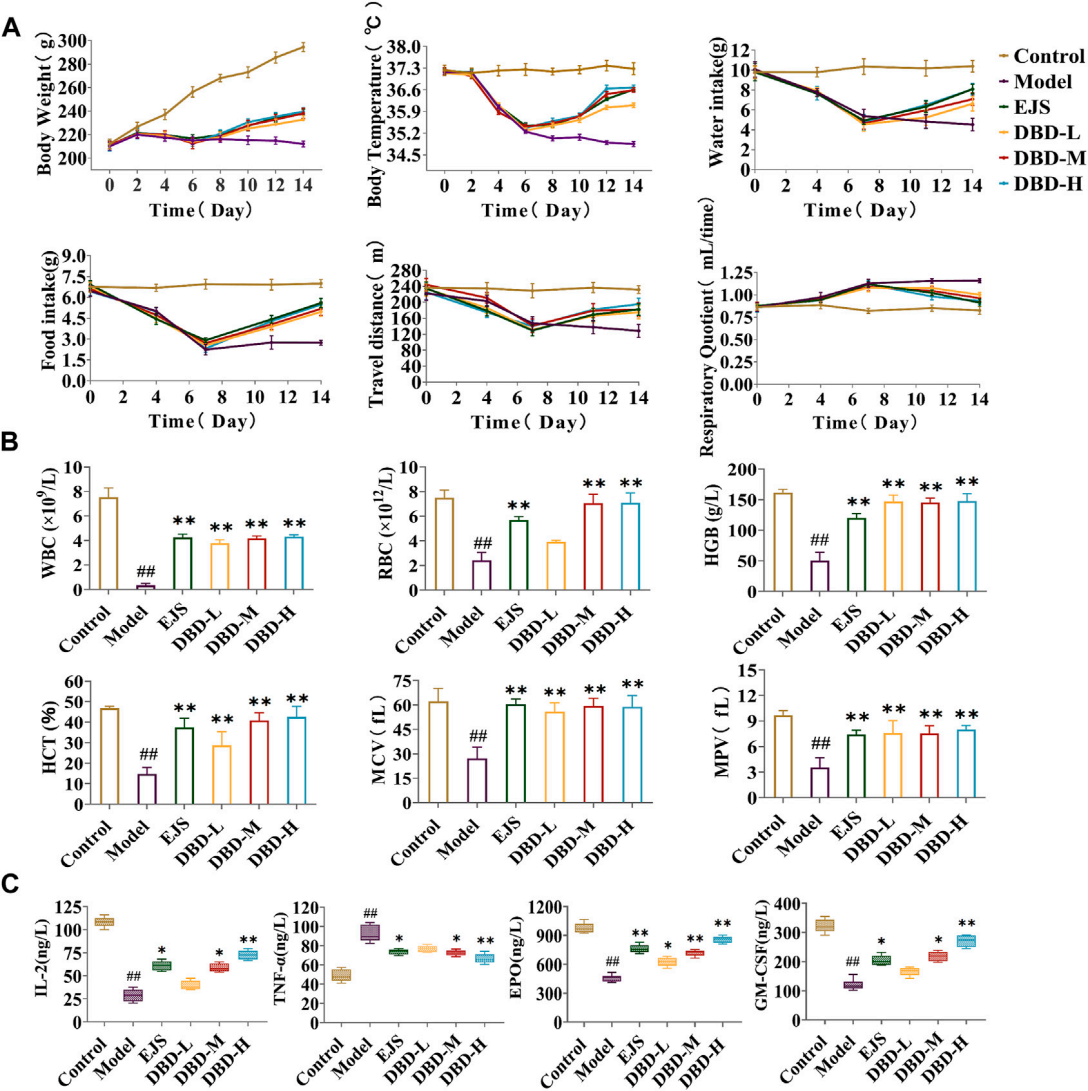
Behavioral, energy metabolism, and biochemical analysis for the evaluation of DBD in BDS rats. **(A)** Body weight, body temperature, water intake, food intake, travel distance, and respiratory quotient in energy metabolism detection. **(B)** The level of HGB, MCV, RBC, HCT, MPV, and WBC in peripheral blood. **(C)** The level of IL-2, TNF-α, EPO, and GM-CSF in serum. ^#^ *p* < 0.05, ^##^ *p* < 0.01 vs. control group. ^*^ *p* < 0.05, ^**^ *p* < 0.01 vs. model group.

**FIGURE 7:**
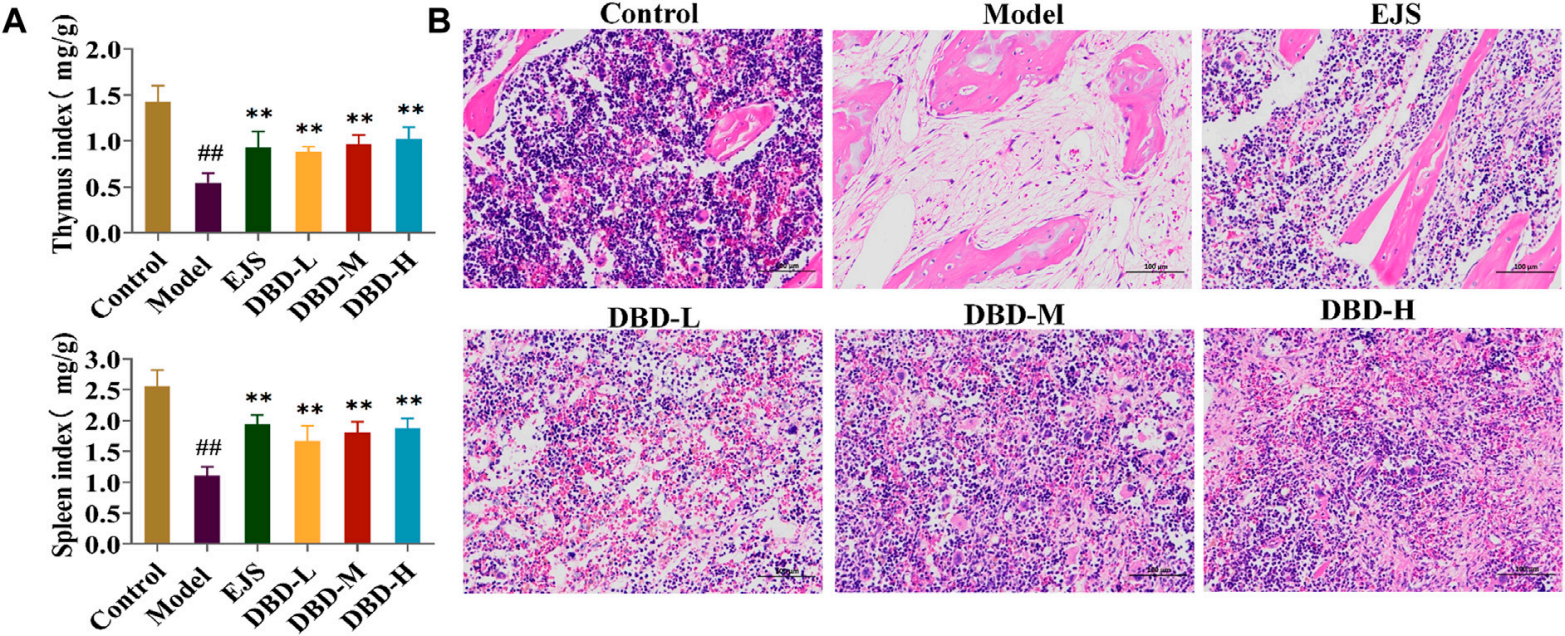
Pathological analysis for the evaluation of DBD in BDS rats. **(A)** The thymus index and spleen index. **(B)** Results of the histopathology analysis of the rat marrow stained with eosin and hematoxylin (×200). ^#^ *p* < 0.05, ^##^ *p* < 0.01 vs. control group. ^*^ *p* < 0.05, ^**^ *p* < 0.01 vs. model group.

During treatment, DBD effectively alleviated weight and body temperature loss, with significant increases in water and food intake and travel distance, as well as a decreased respiratory quotient (Figure 6A). The rats in each treatment group showed varying degrees of recovery in terms of eyes, ears, and paw color. Furthermore, peripheral blood test results, including HCT, MPV, WBC, HGB, RBC, and MCV values, improved (Figure 6B). DBD-M and DBD-H also corrected the aberrant levels of BDS-related biochemical markers (Figure 6C) and increased spleen and thymus indices (Figure 7A). Pathological analysis revealed increased proliferation of hematopoietic cells in the bone marrow (Figure 7B). In conclusion, the immune and hematopoietic functions of rats with BDS improved after DBD treatment. The dose-dependent effects of DBD-L, DBD-M, and DBD-H were comparable or superior to those of Ejiao syrup.

### 3.5 Effects of DBD on the urinary and serum metabolism of rats in the BDS model

**FIGURE 8:**
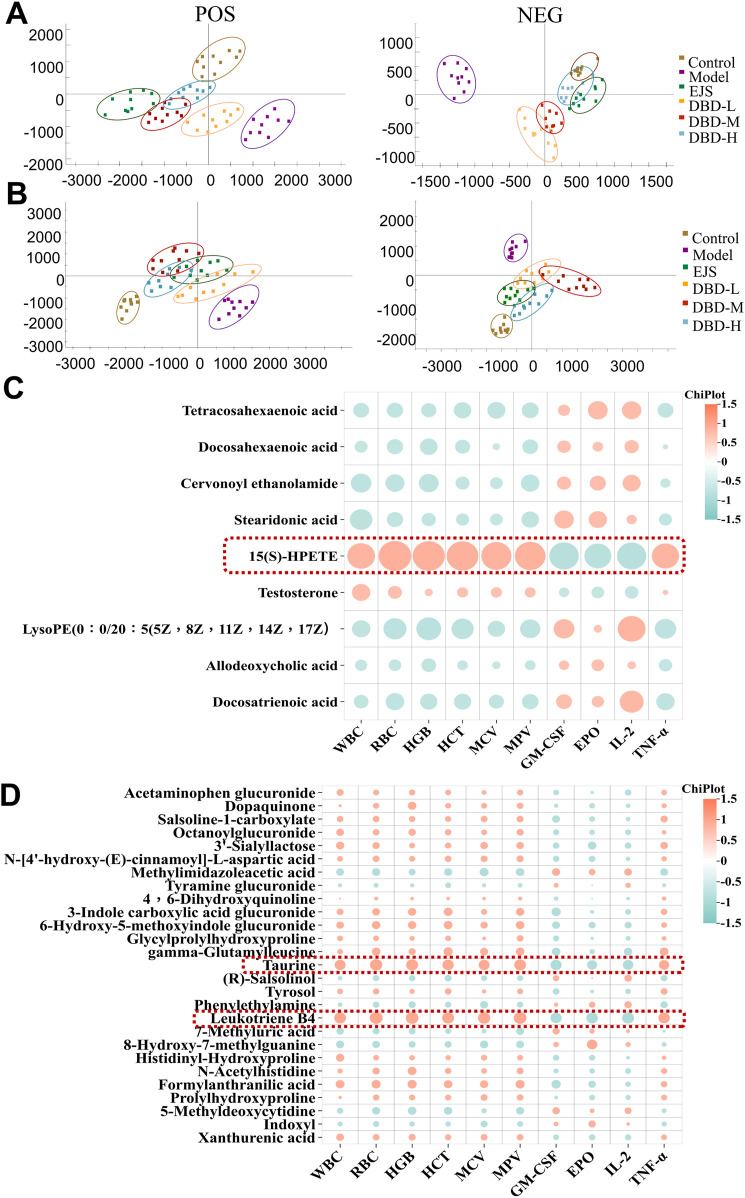
Multivariate statistical analysis of serum and urine metabolites after DBD treatment. **(A)** Serum PCA score plots in ESI^+^ and ESI^−^ modes. **(B)** Urine PCA score plots in ESI^+^ and ESI^−^ modes. **(C)** Correlation analysis between significantly altered serum biomarkers and biochemical indices (https://www.chiplot.online/). **(D)** Correlation analysis between significantly altered urine biomarkers and biochemical indices (https://www.chiplot.online/).

PCA plots of the serum and urine samples showed that the DBD group had clusters similar to those of the control group and different from those of the BDS model group (Figures 8A, B), suggesting that DBD ameliorated the aberrant metabolic network of the BDS model. DBD administration resulted in the normalization of 12 serum and 30 urine BDS biomarkers but significantly altered 9 serum and 27 urine biomarkers (Supplementary Figures S5, S6). These changes restored the metabolic imbalance in essential pathways, such as taurine and hypotaurine, arachidonic acid, phenylalanine, tryptophan, tyrosine, alpha-linolenic acid, pentose and glucuronate interconversion, steroid hormone biosynthesis, and unsaturated fatty acid biosynthesis, thereby effectively treating acetylphenylhydrazine and cyclophosphamide-induced BDS in the rats (Supplementary Figure S7; Supplementary Table S6).

**FIGURE 9:**
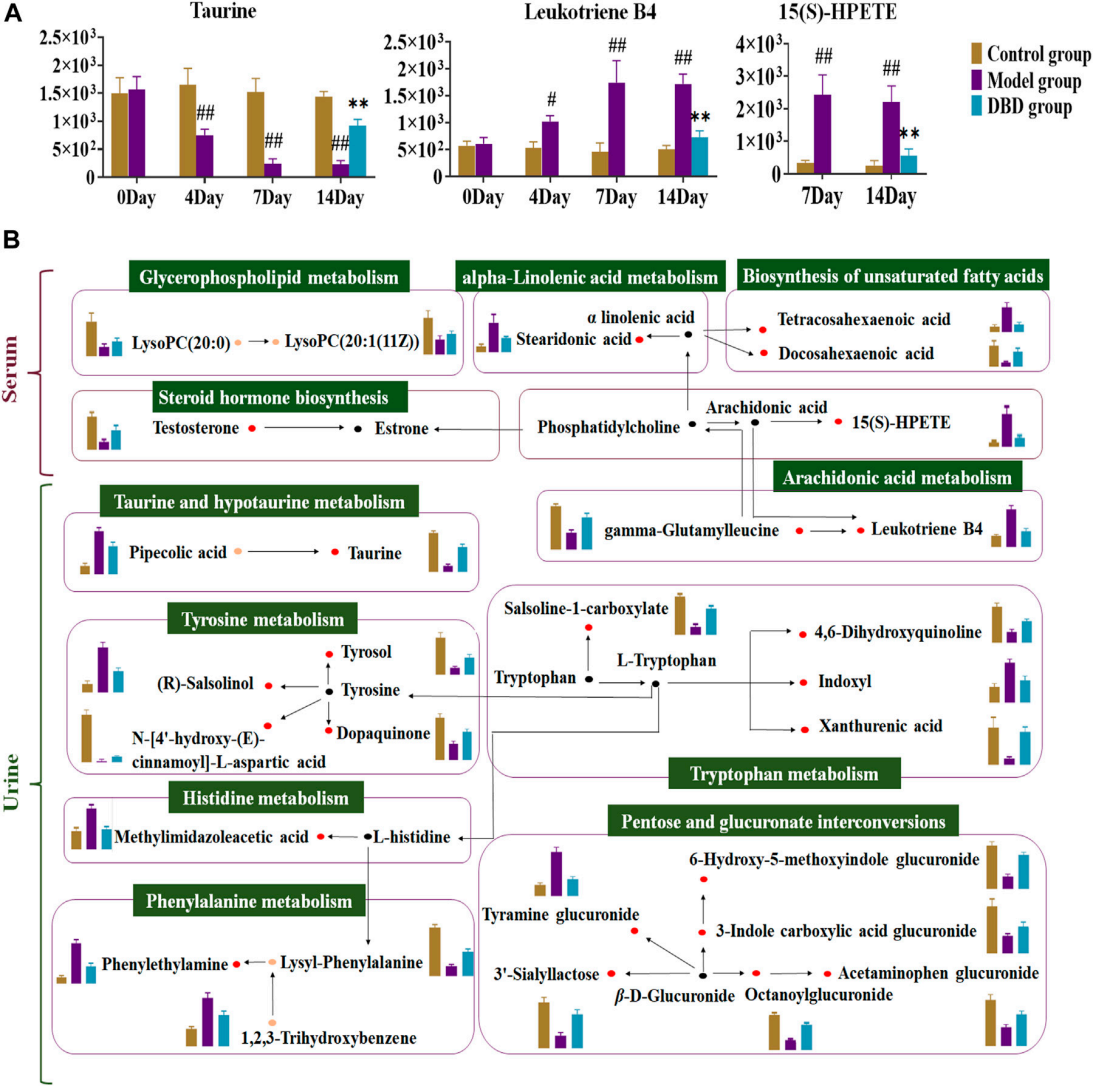
Analysis of DBD’s metabolic pathway and mechanism on potential biomarkers in BDS rats. **(A)** Dynamic change map of core biomarkers. **(B)** The detailed mechanism of DBD in the therapeutic approach to BDS. The red dot represents potentially significant biomarkers in both serum and urine, while the orange dot denotes discrepant potential biomarkers between the two. The black dot designates metabolites connected with the metabolic pathway, and the green box labels the corresponding metabolic pathway. In the column diagram, control’s group is represented by brown; model’s group is represented by purple; and DBD-H’s group is represented by blue. ^#^ *p* < 0.05, ^##^ *p* < 0.01 vs. control group. ^*^ *p* < 0.05, ^**^ *p* < 0.01 vs. model group.

Correlation analysis revealed correlations between 9 significantly altered serum and 27 urinary biomarkers and biochemical indices. Finally, 15(S)-HPETE, leukotriene B4 (LTB4), and taurine were validated as major biomarkers of DBD efficacy (Figures 8C, D). These biomarkers showed significant changes throughout the experiment (Figure 9A). In addition, arachidonic acid metabolism and taurine and hypotaurine metabolism, which are involved in the three crucial biomarkers, were considered key pathways. A metabolic network diagram was constructed to illustrate the metabolic pathways and biomarkers associated with DBD treatment of BDS (Figure 9B).

### 3.6 Characterization of the effective constituents of DBD for treating BDS

UPLC-G2-Si/MS^E^ was used to acquire and characterize the constituent constituents of DBD before and after it exited the bloodstream. A total of 70 chemical compounds present in DBD were evaluated *in vitro,* with a 24 min acquisition time in the positive and negative cation monitoring modes, including 38 positive and 32 negative ions (Supplementary Table S7). Of these, 45 compounds were derived from *Astragali radix*, 22 from *Angelicae sinensis radix*, and 3 compounds from both.

**FIGURE 10:**
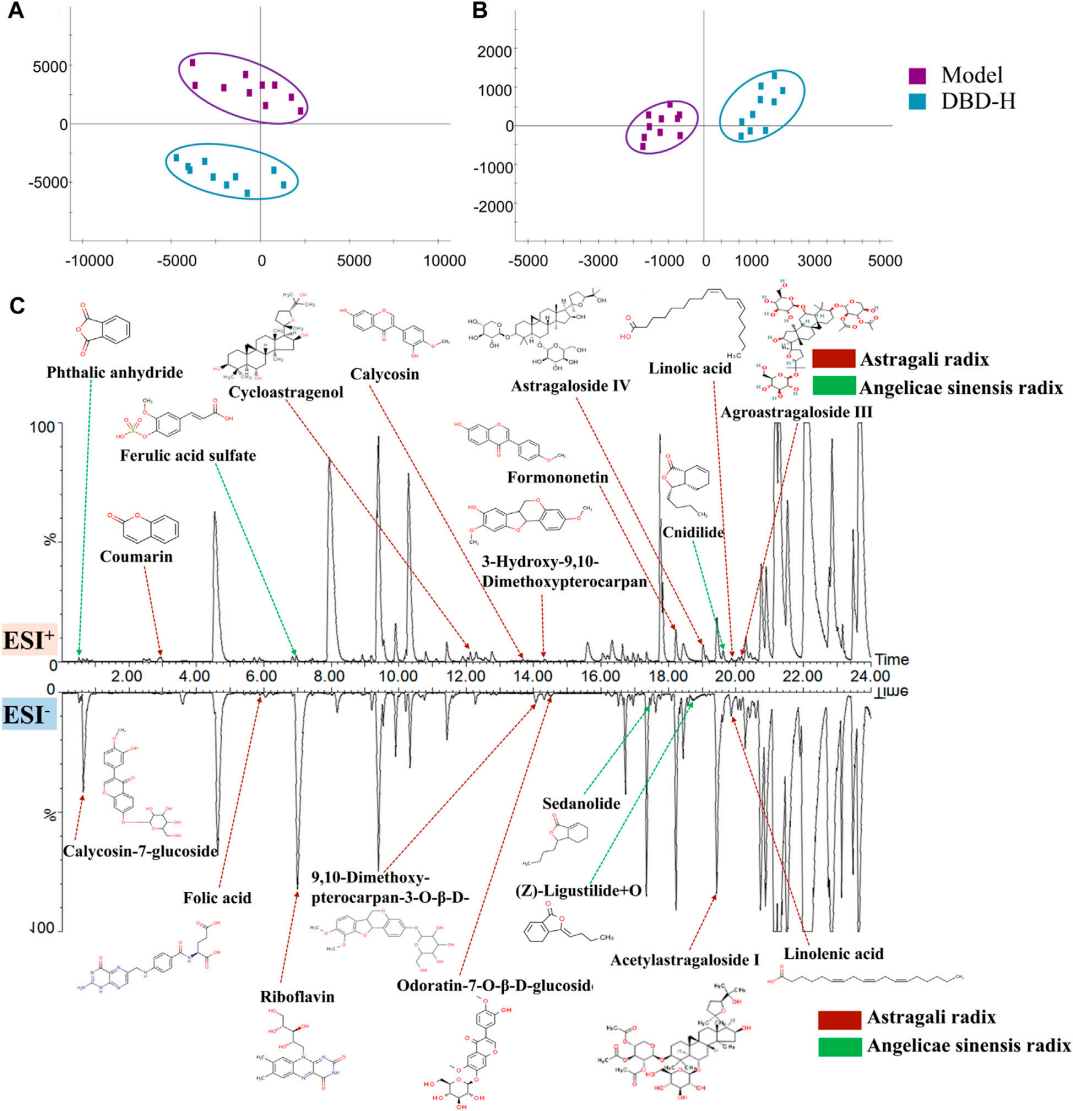
Identification of DBD *in vivo* ingredients is based on PCA. **(A)** and **(B)** PCA score plots were generated for the model and DBD group separately for ESI^+^ and ESI^−^ modes. **(C)** Serum chromatogram of BPI rats with characteristic *in vivo* constituents from DBD *versus* BDS. The compounds indicated by red arrows were sourced from Astragali radix or its metabolites, while those indicated by green arrows were obtained from Angelicae sinensis radix or its metabolites.

Multiple statistical analyses (Figures 10A, B) of the model and DBD groups were performed using the Progenesis QI software to investigate the constituents and metabolic products of DBD. Ultimately, 17 prototype compounds and 3 metabolites were identified as constituents of DBD *in vivo* (Supplementary Table S8). These included astragaloside IV, ferulic acid sulfate, calycosin-7-glucoside, cycloastragenol, and others, which were determined via chromatographic analysis of serum samples utilizing positive and negative ion enrichment methods (Figure 10C).

### 3.7 Correlation analysis between absorbed constituents and biomarkers

**FIGURE 11:**
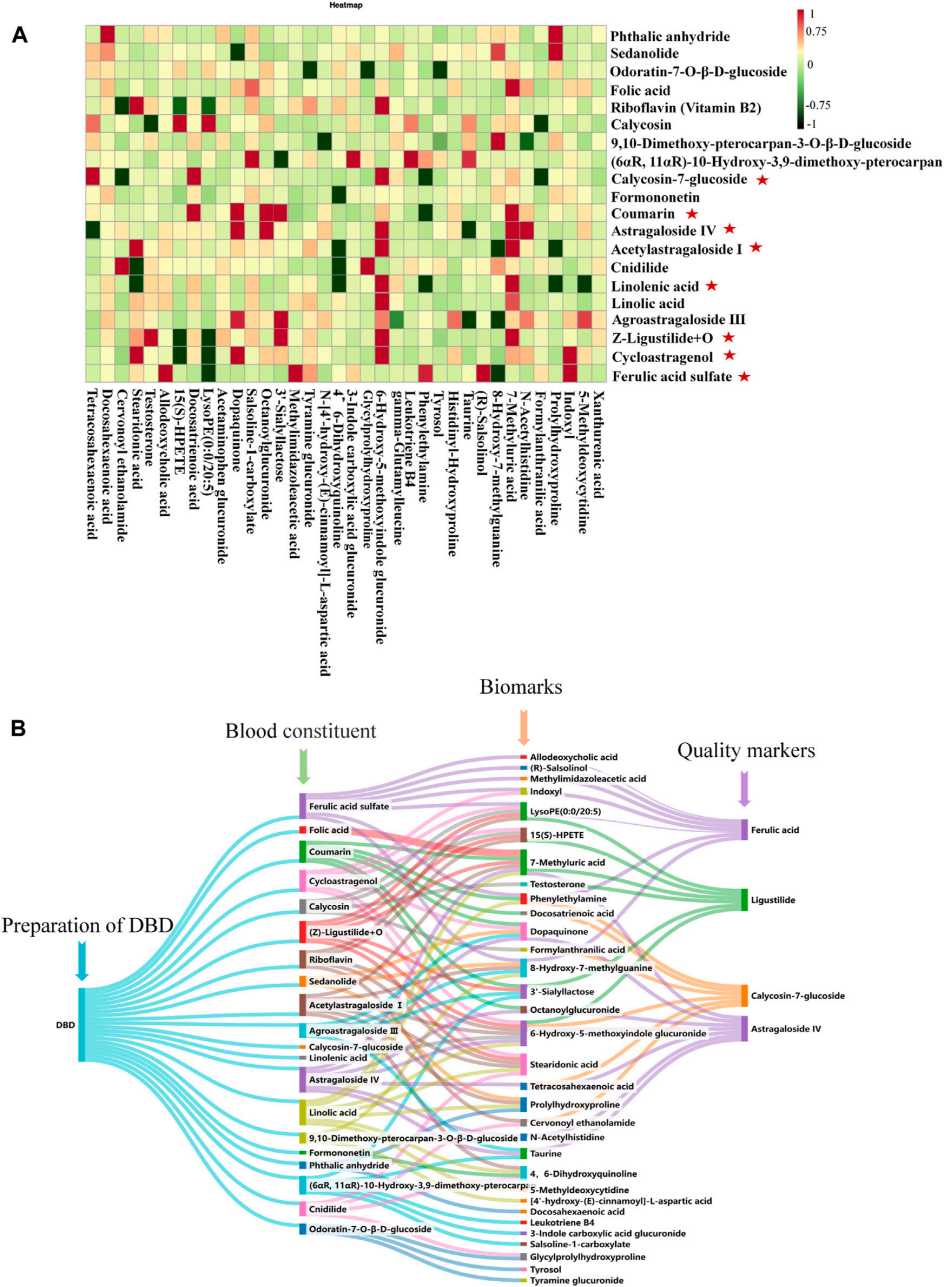
Analysis of effective ingredients and quality markers of DBD. **(A)** Analysis of the correlation between BDS components and serum and urine biomarkers in rats given DBD orally.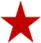: effective ingredients. **(B)** Analysis of the correlation among blood components, biomarkers, and quality markers.

The PCMS analytical model validated the potent therapeutic compounds in DBD that combat BDS. 8 compounds were validated as potential pharmacodynamic constituents underlying the therapeutic effects of DBD in BDS treatment (Figure 11A): calycosin-7-glucoside, coumarin, ferulic acid sulfate, cycloastragenol, (Z)-ligustilide + O, astragaloside IV, acetylastragaloside I, and linoleic acid (Supplementary Table S9).

### 3.8 Identification of DBD quality markers

The levels of coumarin and linoleic acid in DBD were found to be minimal and not measurable, whereas those of astragaloside IV, ferulic acid, and ligustilide, namely, cycloastragenol, acetylastragaloside I, ferulic acid sulfate, and (Z)-ligustilide + O, were poorly accessible. By contrast, prototype compounds are more easily testable, accessible, and stable. Consequently, calycosin-7-glucoside, ferulic acid, ligustilide, and astragaloside IV were identified as quality markers of DBD (Figure 11B).

## 4 Discussion

DBD, a well-established TCM prescription for the treatment of BDS, has recognized hematopoietic effects (Shi et al., 2014). In the present study, we confirmed the efficacy of DBD in treating BDS by observing improvements in metrics such as body weight, body temperature, general status, energy metabolism indices, serum biochemistry levels, peripheral blood profiles, and pathological tissue changes in a rat model of BDS.

In this study, we assessed the metabolic characteristics of a rat model of BDS and examined the effects of DBD using a nonspecific metabolomics methodology. Serum and urine metabolomics revealed 14 serum and 39 urine metabolites with altered expression levels that were primarily involved in amino acid, carbohydrate, and lipid metabolism. According to our results, the mechanism of DBD treatment was linked to 36 significant alterations in biomarkers (9 serum and 27 urine) and 11 related metabolic pathways in the BDS model. Through the correlation of serum and urine biomarkers with biochemical parameters, 15(S)-HPETE, LTB4, and taurine, as well as their metabolism, arachidonic acid, taurine, and hypotaurine metabolism, were identified as the key markers and pathways affected by DBD. Taurine, a free amino acid, has a significant impact on hemoglobin levels, white blood cell levels, immunity in the circulatory system, and hemolytic anemia (Bertolone et al., 2020; Zhang et al., 2022). Our results were consistent with these previous findings; in the present study, DBD regulated taurine expression in the model group, thereby promoting enhanced hematopoietic function.

Oxidation of arachidonic acid to LTB4 by lipoxygenase triggers physiological effects such as increased blood thickness and enhanced cellular activity (Wang et al., 2023). In cases of blood deficiency, increased LTB4 levels in rats disrupt routine peripheral blood indices and impair blood cell function, suggesting weakened hematopoietic activity to some extent (Walters et al., 2021). Furthermore, LTB4 also dysregulates immune function by enhancing TNF-α production (Goldman et al., 1993). Disruption of immune function could lead to moderate nuclear proliferation, which adversely affects the hematopoietic system (Matarraz et al., 2011). 15(S)-HPETE, a polyunsaturated fatty acid, significantly regulates leukocyte function and platelet activation. In addition to inhibiting the production of aortic microsomes and endothelial cells, it promotes eosinophil apoptosis, decreases IL-2 production, and ultimately compromises immune and hematopoietic functions (Huang et al., 1999; Soumya et al., 2014; Quintal Martínez et al., 2023). Our results were consistent with these previous findings.

This study revealed that the mechanism of DBD in treating BDS was also heavily reliant on carbohydrate, lipid, and amino acid metabolism, in addition to the two core pathways previously mentioned. The metabolic pathways associated with histidine, tyrosine, and phenylalanine tended to normalize after oral administration of DBD in the rat model. Methylimidazole acetic acid was produced by histidine methylation, and prolonged dysregulation could lead to abnormalities such as decreased plasma protein levels and blood cell volume (Ackermann et al., 2023). Rats in the BDS model group showed a considerable increase in methylimidazolacetic acid; however, DBD treatment reduced levels closer to those in the control group. Lipid metabolism also demonstrated a sizable tendency to be regulated. Salsoline-1-carboxylate and indoxyl groups play important roles in tryptophan metabolism. Studies have reported that indoxyl-cultured mesenchymal stromal cells could improve angiogenesis, restore vascular integrity, and ameliorate limb ischemia (Bian et al., 2023). Our results demonstrated a significant reversal in salsoline-1-carboxylate and indoxyl expression levels across all DBD treatment groups, indicating a tendency towards the restoration of tryptophan metabolism associated with immune function. Furthermore, abnormalities in the pentose and glucuronate interconversion pathways lower the levels of inflammatory factors and insulin resistance in the bloodstream, and the metabolic function of the pathway improves when inflammatory factor levels are reduced, thereby modulating immune function (Wang et al., 2016; Jain et al., 2018).

To identify the active constituents in DBD, this study used a Chinmedomics strategy to examine the association between absorbed DBD constituents and putative biomarkers in the rat model of BDS, emphasizing the significance of specific compounds in controlling BDS-related metabolic processes and markers. Eight constituents of DBD were identified as potentially effective constituents in BDS. Calycosin-7-glucoside protects the blood-brain barrier, dilates blood vessels, and improves blood microcirculation. Its main mechanism of action involves the enhancement of ischemia and hypoxia by regulating the PI3K/AKT pathway (Chu et al., 2022). Coumarin has anti-heart failure and anti-myocardial ischemic effects, reduces myocardial oxygen consumption, and improves immunity, among other biological activities (Xu et al., 2023). Because of the structural similarity between coumarin and vitamin K, it blocks the vitamin K-dependent clotting pathway and acts as an anticoagulant (Annunziata et al., 2020). Unfortunately, the results of this study did not indicate the importance of ferulic acid, but its core metabolite, ferulic sulfate, which reduces platelet activation and promotes arterial flow, has antithrombotic activity (Liu et al., 2018). (Z)-Ligustilide + O is the product of ligustilide oxidation, which could activate p38 MAPK to prevent apoptosis by downregulating Bcl-3 and upregulating Bax expression, thereby supplying blood flow to the brain (Cheng et al., 2020). Cycloastragenol is the main hydrolysis product of astragaloside IV and mainly stimulates BV-2 cells by activating LPS while inhibiting the pro-inflammatory cytokines TNF-α and nuclear factor kappa-light-chain-enhancer of activated B cells (NF-κB) as well as microglia and astrocytes, thus improving the state of blood deficiency in the body (Li et al., 2020; Chen Y. P. et al., 2022). Acetylastragaloside Ⅰ is a modified product of astragaloside acetylation and has various pharmacological activities, such as promoting angiogenesis, reducing myocardial cell apoptosis, and improving cardiac function. It mediates Toll-like receptor 4 (TLR4)/NF-κB signaling to promote myocardial injury and protect against myocardial ischemia (Li et al., 2002; Zang et al., 2020). Linolenic acid promotes platelet activation and exhibits anti-thrombotic activity (Yuan et al., 2022). These eight constituents possess pharmacological properties including antithrombus, myocardial protection, and accelerated angiogenesis, making them potentially effective constituents in DBD for treating BDS. After assessing the measurability, accessibility, and stability of these elements in DBD, calycosin-7-glucoside, ferulic acid, ligustilide, and astragaloside IV were identified as quality markers.

## 5 Conclusion

This study investigated the efficacy and effective constituents of DBD in treating BDS using the Chinmedomics method. The significant advantages of DBD were predominantly ascribed to taurine, LTB4, and 15(S)-HPETE, which were identified as core biomarkers based on their influence on the critical pathways of taurine, hypotaurine, and arachidonic acid metabolism. After conducting a correlation analysis, 8 effective constituents were demonstrated as underlying pharmacodynamic constituents for treating BDS, with calycosin-7-glucoside, ferulic acid, ligustilide, and astragaloside IV later identified as quality markers for DBD. These findings provide support for quality control measures for DBD and promote innovation in medication.

## Data Availability

The raw data supporting the conclusion of this article will be made available by the authors, without undue reservation.
