# Supplementary material for: Chinmedomics strategy for elucidating the effects and effective constituents of Danggui Buxue Decoction in treating blood deficiency syndrome
Source: Front Mol Biosci. 2024 Mar 15;11:1376345. doi: 10.3389/fmolb.2024.1376345 (PMC10978583; doi:10.3389/fmolb.2024.1376345)
Supplement: Supplementary file 1 [file DataSheet1.docx]

Supplementary Material

# Supplementary Figures and Tables

## Supplementary Figures


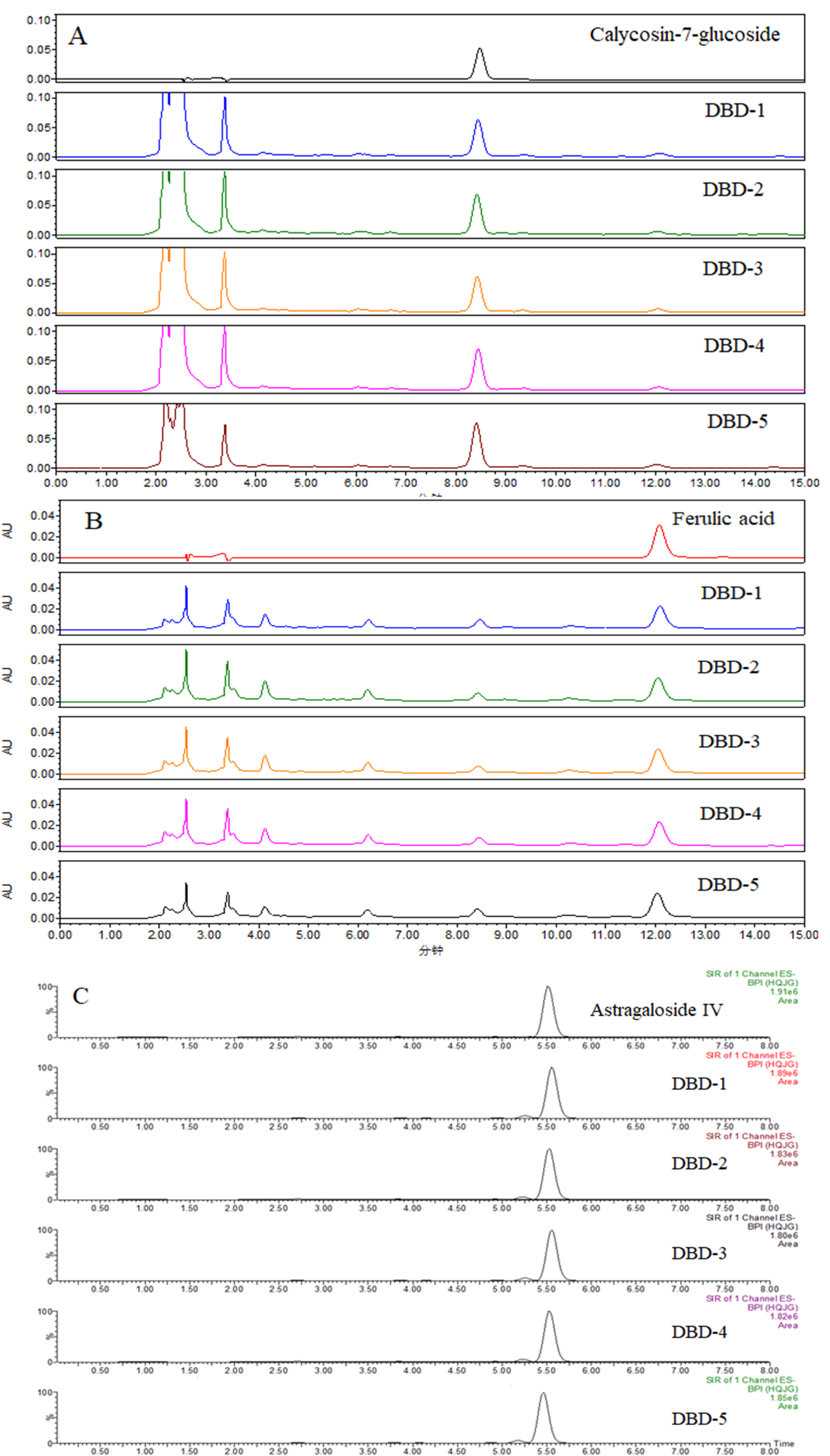


**Supplementary Figure S1.** Content determination of DBD.


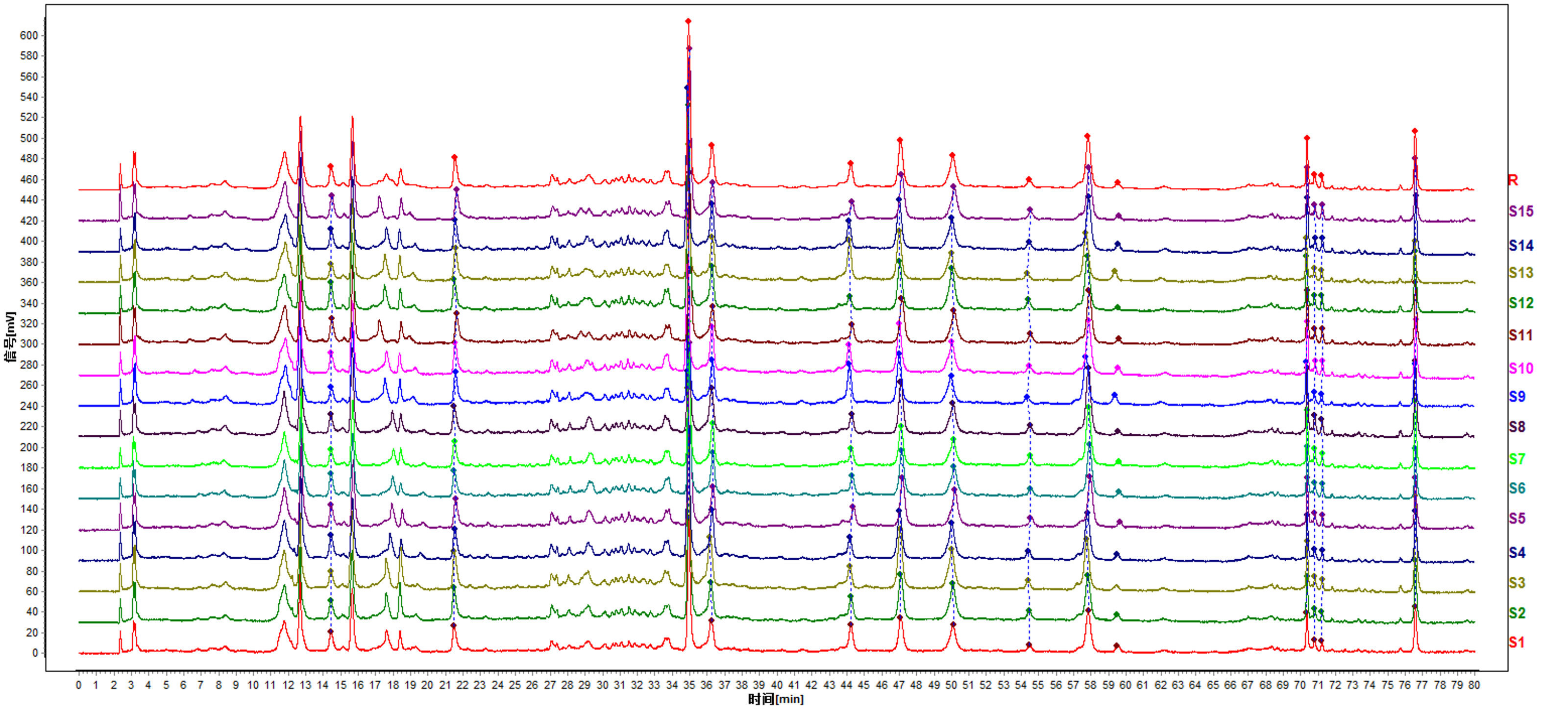


**Supplementary Figure S2.** Fingerprint of 15 batches of DBD.


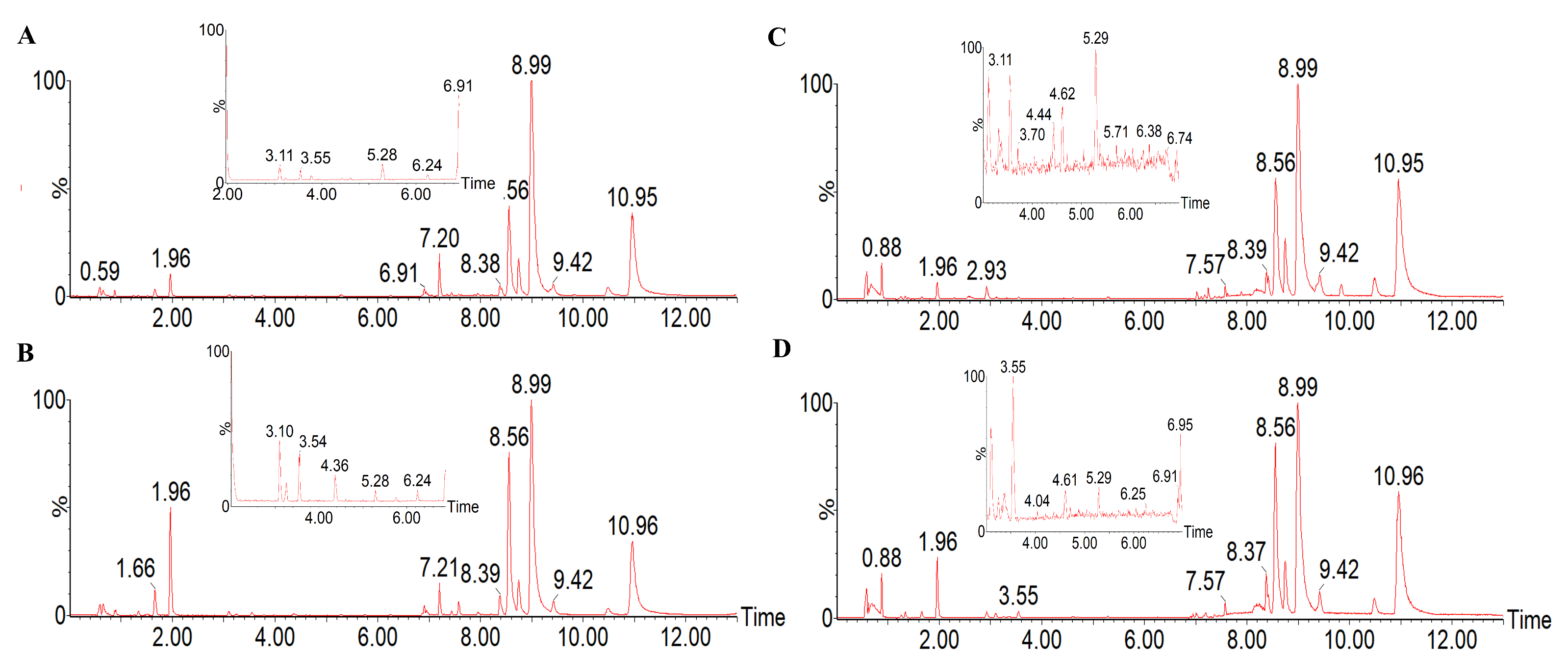


**Supplementary Figure S3.** The control and model rat serum chromatogram (in proper order which from up to down) in positive mode (A-B) and negative mode (C-D) respectively.


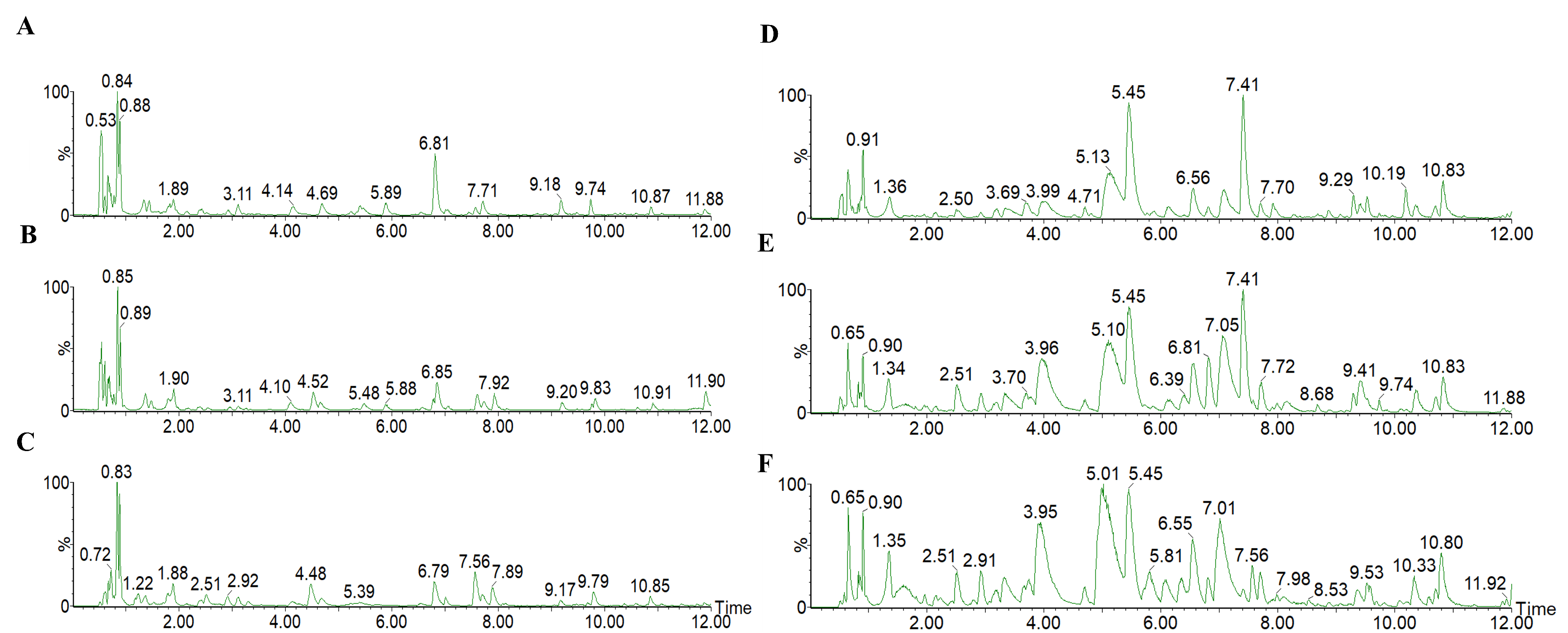


**Supplementary Figure S4.** The 0,4, 7 days model urine chromatogram (in proper order which from up to down) in positive mode (A-C) and negative mode (D-E) respectively.


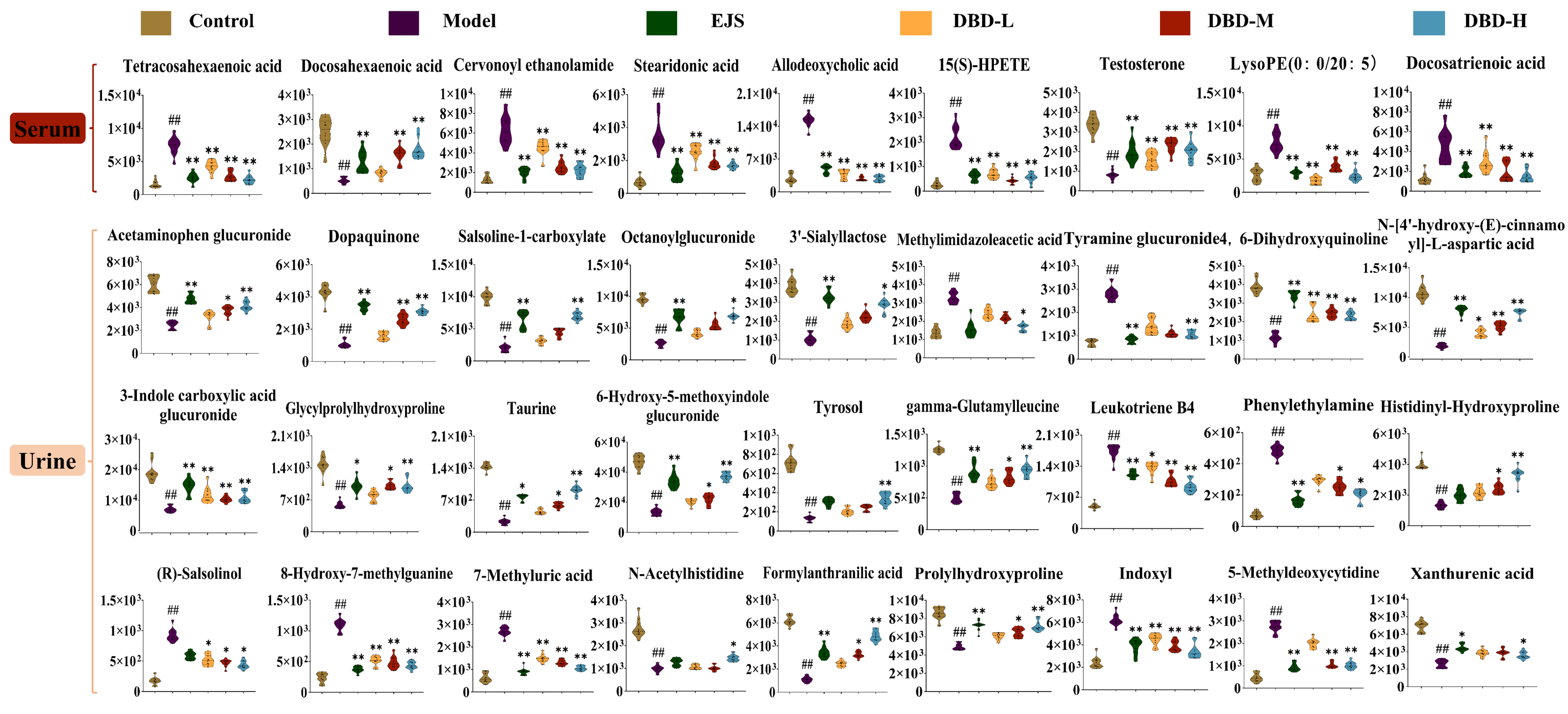


**Supplementary Figure S5.** Comparative strength for potential biomarkers in urine and serum for the control, model, DBD and EJS groups. #*p* < 0.05, ##*p* < 0.01 vs. control group. **p* < 0.05, ***p* < 0.01 vs. model group.


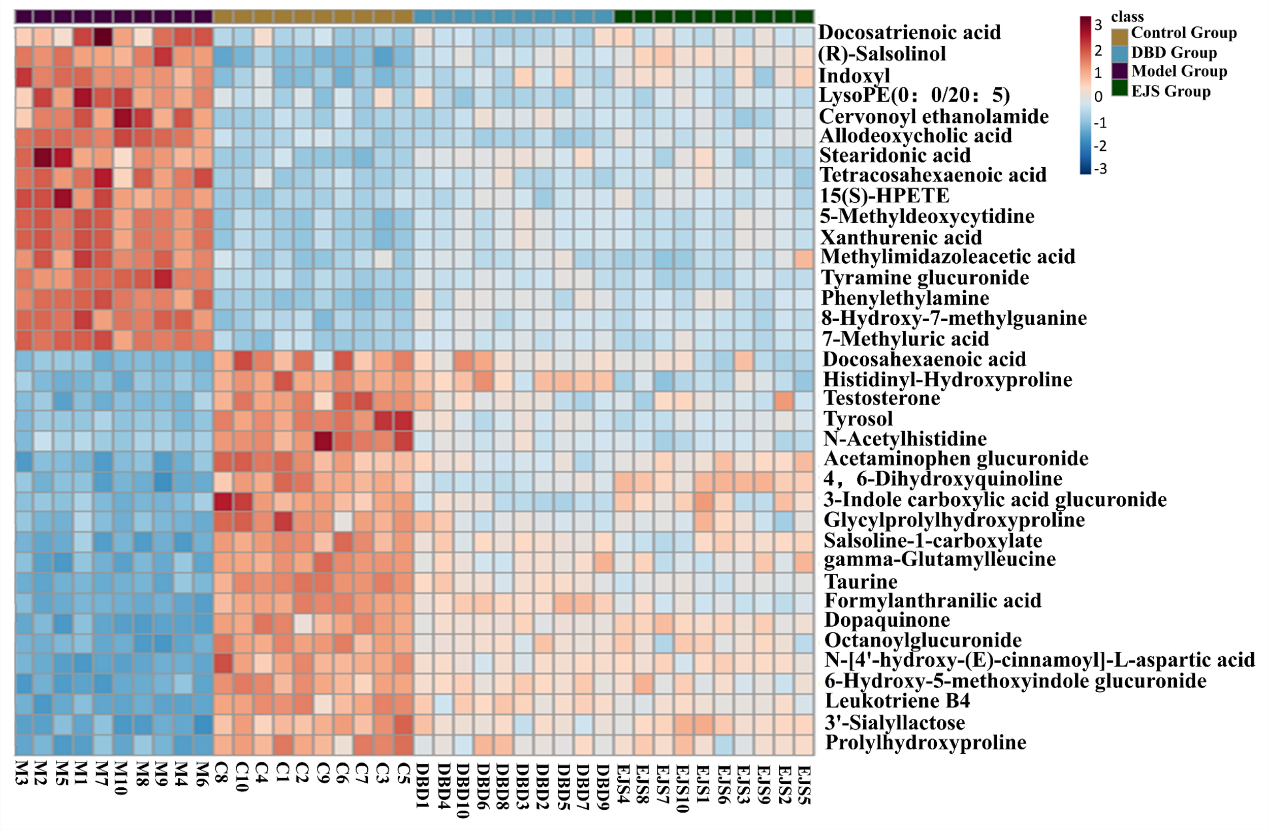


**Supplementary Figure S6.** Based on the potential significant candidates that were retrieved using OPLS-DA analysis, the heatmaps were created. Rows: groups; columns: metabolites; Distinct discernible variations among the control, model, EJS, and DBD groups.


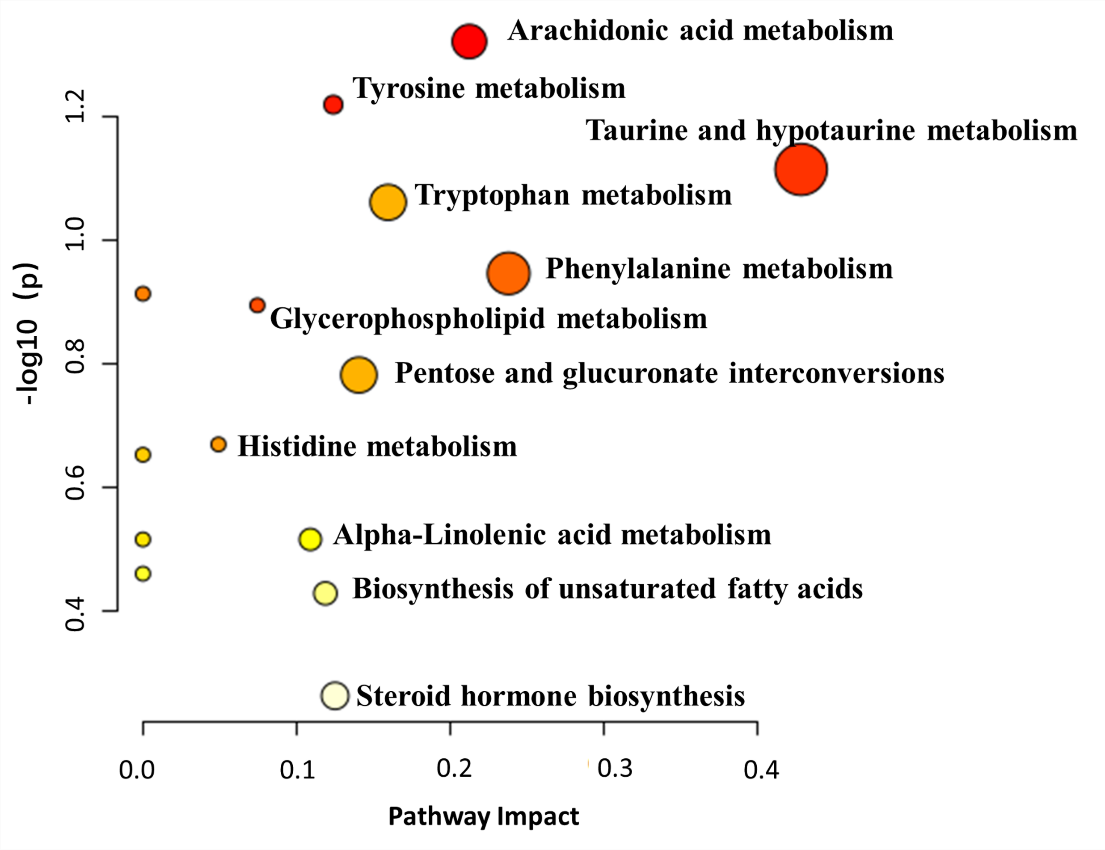


**Supplementary Figure S7.** Based on the potential significant candidates that were retrieved using OPLS-DA analysis, the heatmaps were created. Rows: groups; columns: metabolites; Distinct discernible variations among the control, model, EJS, and DBD groups.

## Supplementary Tables

**Supplementary Table S1.** Content determination results in five batches of DBD

| **Component** | **Batch number** | **Content（mg/g）** | **Average（mg/g）** | **Content RSD（%）** |
| --- | --- | --- | --- | --- |
| Calycosin-7-glucoside | 1 | 1.191 | 1.186 | 0.35 |
|  | 2 | 1.182 |  |  |
|  | 3 | 1.182 |  |  |
|  | 4 | 1.186 |  |  |
|  | 5 | 8.596 |  |  |
| Astragaloside IV | 1 | 1.829 | 1.810 | 0.59 |
|  | 2 | 1.807 |  |  |
|  | 3 | 1.805 |  |  |
|  | 4 | 1.806 |  |  |
|  | 5 | 1.803 |  |  |
| Ferulic acid | 1 | 0.210 | 0.213 | 1.74 |
|  | 2 | 0.215 |  |  |
|  | 3 | 0.209 |  |  |
|  | 4 | 0.211 |  |  |
|  | 5 | 0.218 |  |  |

**Supplementary Table S2.** Fingerprint similarity of 15 batches of DBD

| Batch number | S1 | S2 | S3 | S4 | S5 | S6 | S7 | S8 | S9 | S10 | S11 | S12 | S13 | S14 | S15 |
| --- | --- | --- | --- | --- | --- | --- | --- | --- | --- | --- | --- | --- | --- | --- | --- |
| similarity (control) | 0.979 | 0.988 | 0.986 | 0.989 | 0.989 | 0.985 | 0.982 | 0.973 | 0.920 | 0.964 | 0.986 | 0.993 | 0.988 | 0.978 | 0.931 |

**Supplementary Table S3.** Specific information table of potential biomarkers in the rat model of BDS

| **NO.** | **Rt** | **M/Z** | **Identification** | **Formular** | **Adducts** | **Fragment** | **Source** | **Trend** |
| --- | --- | --- | --- | --- | --- | --- | --- | --- |
| 1 | 0.63 | 300.0345 | N-Acetylglucosamine 6-sulfate | C_8_H_15_NO_9_S | M-H | 243；212；198；170；124 | Urine | ↓** |
| 2 | 0.67 | 138.0504 | Methylimidazoleacetic acid | C_6_H_8_N_2_O_2_ | M+H | 123；107；95；83 | Urine | ↑* |
| 3 | 0.71 | 632.1992 | 3'-Sialyllactose | C_23_H_39_NO_19_ | M-H | 352；300；255；194；96 | Urine | ↓** |
| 4 | 0.74 | 198.0882 | N-Acetylhistidine | C_8_H_11_N_3_O_3_ | M+H | 180；141；102 | Urine | ↓** |
| 5 | 0.84 | 212.0999 | Prolylhydroxyproline | C_10_H_16_N_2_O_4_ | M+H | 153；109；95 | Urine | ↓* |
| 6 | 0.91 | 160.1328 | DL-2-Aminooctanoic acid | C_8_H_17_NO_2_ | M+H | 143；126 | Urine | ↑** |
| 7 | 1.00 | 286.1386 | Glycylprolylhydroxyproline | C_12_H_19_N_3_O_5_ | M+H | 270；244；172；130 | Urine | ↓* |
| 8 | 1.04 | 269.1275 | Histidinyl-Hydroxyproline | C_11_H_16_N_4_O_4_ | M+H | 211；200；181；164；158 | Urine | ↓* |
| 9 | 1.10 | 261.1424 | gamma-Glutamylleucine | C_11_H_20_N_2_O_5_ | M+H | 245；226；203；134；98 | Urine | ↓* |
| 10 | 1.12 | 314.1255 | Tyramine glucuronide | C_14_H_19_NO_7_ | M+H | 226；138；121；103；91 | Urine | ↑** |
| 11 | 1.21 | 229.1529 | Leucylproline | C_11_H_20_N_2_O_3_ | M+H | 166；127；84 | Urine | ↑** |
| 12 | 1.39 | 174.1124 | Tetracosahexaenoic acid | C_24_H_36_O_2_ | M+Na | 184；125；104；87 | Serum | ↑** |
| 13 | 2.64 | 328.0389 | Adenosine 2',3'-cyclic phosphate | C_10_H_12_N_5_O_6_P | M-H | 215；134 | Urine | ↓** |
| 14 | 1.90 | 182.0674 | 8-Hydroxy-7-methylguanine | C_6_H_7_N_5_O_2_ | M+H | 168；152；127；110 | Urine | ↑** |
| 15 | 1.94 | 242.1129 | 5-Methyldeoxycytidine | C_10_H_15_N_3_O_4_ | M+H | 222；182；138；108 | Urine | ↑** |
| 16 | 2.31 | 194.0434 | Dopaquinone | C_9_H_9_NO_4_ | M-H | 157；141；125 | Urine | ↓* |
| 17 | 2.58 | 166.0472 | Formylanthranilic acid | C_8_H_7_NO_3_ | M+H | 148；120 | Urine | ↓** |
| 18 | 2.82 | 130.0383 | Pipecolic acid | C_6_H_11_NO_2_ | M+H | 112；86 | Urine | ↑* |
| 19 | 2.90 | 183.0518 | 7-Methyluric acid | C_6_H_6_N_4_O_3_ | M+H | 168；154；136；124 | Urine | ↑** |
| 20 | 3.40 | 127.0365 | 1,2,3-Trihydroxybenzene | C_6_H_6_O_3_ | M+H | 116；103；91；70 | Urine | ↑* |
| 21 | 3.78 | 294.1774 | Lysyl-Phenylalanine | C_15_H_23_N_3_O_3_ | M+H | 244；220；201；122 | Urine | ↓* |
| 22 | 4.07 | 279.1494 | L-phenylalanyl-L-hydroxyproline | C_14_H_18_N_2_O_4_ | M+H | 243；184；120；103；91；77 | Urine | ↓** |
| 23 | 4.51 | 162.0526 | 4,6-Dihydroxyquinoline | C_9_H_7_NO_2_ | M+H | 144；116 | Urine | ↓* |
| 24 | 4.78 | 276.0551 | N-[4'-hydroxy-(E)-cinnamoyl]-L-aspartic acid | C_13_H_13_NO_6_ | M-H | 230；204；186 | Urine | ↓** |
| 25 | 5.14 | 206.0431 | Xanthurenic acid | C_10_H_7_NO_4_ | M+H | 188；178；160；132 | Urine | ↓** |
| 26 | 5.2 | 355.2619 | Adrenic acid | C_22_H_36_O_2_ | M+H | 301；287；237；219；203 | Serum | ↑** |
| 27 | 5.39 | 333.2009 | Testosterone | C_19_H_28_O_2_ | M-H | 158 | Serum | ↓** |
| 28 | 5.39 | 299.1987 | Stearidonic acid | C_18_H_28_O_2_ | M+H | 192；177；136 | Serum | ↑** |
| 29 | 5.60 | 190.0507 | Kynurenic acid | C_10_H_7_NO_3_ | M+H | 172；149 | Urine | ↓** |
| 30 | 5.70 | 326.0838 | Acetaminophen glucuronide | C_14_H_17_NO_8_ | M-H | 326；269；175；150；113；99 | Urine | ↓** |
| 31 | 5.75 | 373.2691 | Docosatrienoic acid | C_22_H_38_O_2_ | M+H | 281；184；77 | Serum | ↑** |
| 32 | 5.97 | 134.0597 | Indoxyl | C_8_H_7_NO | M+H | 133 | Urine | ↑** |
| 33 | 6.46 | 338.0801 | 3-Indole carboxylic acid glucuronide | C_15_H_15_NO_8_ | M+H | 189；164 | Urine | ↓** |
| 34 | 6.57 | 126.0245 | Taurine | C_2_H_7_NO_3_S | M+H | 118；99 | Urine | ↓** |
| 35 | 6.76 | 335.2206 | 15(S)-HPETE | C_20_H_32_O_4_ | M-H | 303；285 | Serum | ↑** |
| 36 | 6.98 | 338.0818 | 6-Hydroxy-5-methoxyindole glucuronide | C_15_H_17_NO_8_ | M-H | 250；162；148；144 | Urine | ↓** |
| 37 | 6.99 | 357.2764 | Cervonoyl ethanolamide | C_24_H_36_O_3_ | M+H | 319；253；239；225；184；161 | Serum | ↑** |
| 38 | 7.04 | 392.2882 | Allodeoxycholic acid | C_24_H_40_O_4_ | M-H | 357；317；265；216 | Serum | ↑** |
| 39 | 7.05 | 242.9953 | Estrone sulfate | C_18_H_22_O_5_S | M-H | 193；175；163 | Urine | ↓** |
| 40 | 7.5 | 255.0627 | 5-L-Glutamyl-taurine | C_7_H_14_N_2_O_6_S | M+H | 237；223；171 | Urine | ↑** |
| 41 | 7.89 | 167.0731 | L-3-Phenyllactic acid | C_9_H_10_O_3_ | M+H | 137；134；123；107；105 | Urine | ↑** |
| 42 | 8.01 | 540.3224 | LysoPE(0:0/20:5(5Z,8Z,11Z,14Z,17Z)) | C_25_H_42_NO_7_P | M-H | 478；452；438；282 | Serum | ↑** |
| 43 | 8.22 | 236.0929 | Salsoline-1-carboxylate | C_12_H_15_NO_4_ | M-H | 204；178 | Urine | ↓** |
| 44 | 8.49 | 510.3522 | LysoPC(17:0) | C_25_H_52_NO_7_P | M+H | 496；327；390；292 | Serum | ↓** |
| 45 | 8.85 | 508.3815 | LysoPC(O-18:0/0:0) | C_26_H_54_NO_6_P | M+H | 355；273；254；161；121；95 | Serum | ↑* |
| 46 | 8.87 | 180.1121 | (R)-Salsolinol | C_10_H_13_NO_2_ | M+H | 162；120；107 | Urine | ↑** |
| 47 | 9.54 | 139.0745 | Tyrosol | C_8_H_10_O_2_ | M+H | 111；107；77 | Urine | ↓* |
| 48 | 9.69 | 357.0953 | Octanoylglucuronide | C_14_H_24_O_8_ | M-H | 269；239；175 | Urine | ↓** |
| 49 | 9.88 | 337.2357 | Leukotriene B4 | C_20_H_32_O_4_ | M+H | 319；301；283；226；201；181；109 | Urine | ↑* |
| 50 | 10.03 | 122.0241 | Phenylethylamine | C_8_H_11_N | M+H | 122；120 | Urine | ↑** |
| 51 | 10.23 | 594.3779 | LysoPC(20:1(11Z)) | C_28_H_56_NO_7_P | M-H | 579；506；309 | Serum | ↓** |
| 52 | 11.65 | 596.3228 | LysoPC(20:0/0:0) | C_28_H_58_NO_7_P | M-H | 536；508；311；283；255；227 | Serum | ↓** |
| 53 | 11.92 | 329.2477 | Docosahexaenoic acid | C_22_H_32_O_2_ | M+H | 347；227；121；95；81 | Serum | ↓* |

Note: "↑↓" increases or decreases the metabolic level in the model; compared with the control group, **P*<0.05, ***P*<0.01.

**Supplementary Table S4.** Serum metabolism pathway analysis of potential biomarker MetPA in rat model of BDS

| **NO.** | | **Pathway name** | **Class** | **Total** | **Expected** | **Hits** | **Raw p** | **-log(p)** | **Impact** |
| --- | --- | --- | --- | --- | --- | --- | --- | --- | --- |
| 1 | Biosynthesis of unsaturated fatty acids | | Lipid metabolism | 36 | 0.29748 | 3 | 0.5531 | 1.1132 | 0.1846 |
| 2 | Arachidonic acid metabolism | | Lipid metabolism | 36 | 0.6671 | 2 | 0.1351 | 0.8694 | 0.1485 |
| 3 | Alpha-Linolenic acid metabolism | | Lipid metabolism | 13 | 1.4024 | 2 | 0.2507 | 0.9522 | 0.1093 |
| 4 | Steroid hormone biosynthesis | | Lipid metabolism | 77 | 1.6361 | 2 | 0.2701 | 0.5686 | 0.1089 |
| 5 | Glycerophospholipid metabolism | | Lipid metabolism | 36 | 0.42497 | 4 | 0.5531 | 1.2952 | 0.0047 |
| 6 | Ether lipid metabolism | | Lipid metabolism | 20 | 0.66417 | 1 | 0.13508 | 0.8694 | 0 |

**Supplementary Table S5.** Urine metabolism pathway analysis of potential biomarker MetPA in rat model of BDS

| **NO.** | **Pathway name** | **Class** | **Total** | **Expected** | **Hits** | **Raw p** | **-log(p)** | **Impact** |
| --- | --- | --- | --- | --- | --- | --- | --- | --- |
| 1 | Taurine and hypotaurine metabolism | Metabolism of other amino acids | 8 | 0.76494 | 3 | 0.0120 | 2.8009 | 0.4286 |
| 2 | Arachidonic acid metabolism | Lipid metabolism | 36 | 0.87118 | 2 | 0.2338 | 1.0377 | 0.2381 |
| 3 | Steroid hormone biosynthesis | Lipid metabolism | 77 | 0.27623 | 1 | 0.2252 | 1.5475 | 0.1749 |
| 4 | Pentose and glucuronate interconversions | Carbohydrate metabolism | 18 | 0.87104 | 7 | 0.3299 | 0.4817 | 0.1806 |
| 5 | Tryptophan metabolism | Lipid metabolism | 41 | 0.59495 | 5 | 0.2351 | 0.4335 | 0.1490 |
| 6 | Phenylalanine metabolism | Amino acid metabolism | 12 | 1.6361 | 4 | 0.1847 | 0.6287 | 0.1197 |
| 7 | Tyrosine metabolism | Amino acid metabolism | 42 | 0.31873 | 4 | 0.6100 | 1.3147 | 0.1053 |
| 8 | Caffeine metabolism | Biosynthesis of other secondary metabolites | 12 | 0.12749 | 1 | 0.2338 | 0.8811 | 0 |
| 9 | Histidine metabolism | Amino acid metabolism | 16 | 0.76494 | 1 | 0.2992 | 0.7240 | 0 |
| 10 | Lysine degradation | Amino acid metabolism | 25 | 0.29748 | 1 | 0.4272 | 0.5693 | 0 |
| 11 | Primary bile acid biosynthesis | Lipid metabolism | 46 | 0.33997 | 1 | 0.6440 | 0.1911 | 0 |

**Supplementary Table S6.** Metabolism pathway analysis of Danggui Buxue Decoction MetPA in serum and urine of BDS rats

| **NO.** | **Pathway name** | **Class** | **Total** | **Expected** | **Hits** | **Raw p** | **-log(p)** | **Impact** |
| --- | --- | --- | --- | --- | --- | --- | --- | --- |
| 1 | Taurine and hypotaurine metabolism | Metabolism of other amino acids | 8 | 0.32647 | 2 | 0.0820 | 1.0866 | 0.4286 |
| 2 | Phenylalanine metabolism | Amino acid metabolism | 12 | 0.79375 | 3 | 0.1205 | 0.9191 | 0.2381 |
| 3 | Arachidonic acid metabolism | Lipid metabolism | 36 | 0.43765 | 3 | 0.0539 | 1.2688 | 0.1997 |
| 4 | Tryptophan metabolism | Lipid metabolism | 41 | 0.21706 | 4 | 0.1755 | 1.0237 | 0.1748 |
| 5 | Pentose and glucuronate interconversions | Carbohydrate metabolism | 18 | 0.40939 | 6 | 0.0726 | 0.7261 | 0.1481 |
| 6 | Steroid hormone biosynthesis | Lipid metabolism | 77 | 0.34514 | 1 | 0.5693 | 0.2447 | 0.1309 |
| 7 | Tyrosine metabolism | Amino acid metabolism | 42 | 0.67138 | 4 | 0.0825 | 1.2159 | 0.1295 |
| 8 | Biosynthesis of unsaturated fatty acids | Lipid metabolism | 36 | 0.24693 | 2 | 0.3922 | 0.4065 | 0.1271 |
| 9 | Alpha-Linolenic acid metabolism | Lipid metabolism | 13 | 0.45147 | 1 | 0.3218 | 0.4967 | 0.1184 |
| 10 | Glycerophospholipid metabolism | Lipid metabolism | 36 | 1.02923 | 2 | 0.1575 | 0.8717 | 0.0792 |
| 11 | Histidine metabolism | Amino acid metabolism | 16 | 0.26074 | 1 | 0.2356 | 0.6948 | 0.0498 |
| 12 | Caffeine metabolism | Biosynthesis of other secondary metabolites | 12 | 0.31947 | 1 | 0.3649 | 0.6759 | 0 |
| 13 | Lysine degradation | Amino acid metabolism | 25 | 0.57592 | 1 | 0.3455 | 0.5216 | 0 |
| 14 | Primary bile acid biosynthesis | Lipid metabolism | 46 | 0.36148 | 1 | 0.2765 | 0.4638 | 0 |

**Supplementary Table S7.** The 70 chemical components which characterized of DBD *in vitro*

| **No** | **Rt** | **Identification** | **Formular** | **Adducts** | **M/z** | **Fragment** | **Origin** |
| --- | --- | --- | --- | --- | --- | --- | --- |
| 1 | 0.61 | Arginine | C_6_H_14_N_4_O_2_ | M+H | 175.1207 | 160 | a |
| 2 | 0.70 | 4-Aminobutanoate | C_4_H_9_NO_2_ | M-H | 102.0567 | 74 | a |
| 3 | 1.04 | alpha-L-Rhamnose | C_6_H_12_O_5_ | M-H | 163.0616 | 147 | a |
| 4 | 1.93 | Nicotinic acid | C_6_H_5_NO_2_ | M-H | 168.0316 | 122；85 | a,b |
| 5 | 3.97 | Proline | C_5_H_9_NO_2_ | M-H | 114.0554 | 101；85 | a |
| 6 | 4.53 | Chinnamic acid | C_16_H_18_O_9_ | M-H | 353.0885 | 293；263；191；135 | b |
| 7 | 5.28 | Folic acid | C_19_H_19_N_7_O_6_ | M-H | 441.1397 | 197；183 | a |
| 8 | 6.11 | Neochlorogenic acid | C_16_H_18_O_9_ | M-H | 353.0882 | 289；191；179；135 | b |
| 9 | 6.35 | Vanillic acid | C_8_H_8_O_4_ | M-H | 168.0409 | 153；135；123 | b |
| 10 | 6.38 | Rutin | C_27_H_30_O_16_ | M+H | 610.1528 | 511； 279；264；239 | a |
| 11 | 6.39 | Chlorogenic acid | C_16_H_18_O_9_ | M-H | 353.0882 | 191；179；135 | b |
| 12 | 6.42 | Phthalic acid | C_8_H_6_O_4_ | M-H | 166.0275 | 121；77 | b |
| 13 | 6.61 | Caffeic acid | C_9_H_8_O_4_ | M-H | 179.0357 | 147；135；117 | a,b |
| 14 | 7.11 | Cryptochloric acid | C_16_H_18_O_9_ | M-H | 353.0882 | 191；179；136 | b |
| 15 | 7.52 | Riboflavin | C_17_H_20_N_4_O_6_ | M+H | 376.1384 | 359；282；243 | a |
| 16 | 7.76 | Isoquercitrin | C_21_H_20_O_12_ | M+H | 465.1053 | 317；161 | a |
| 17 | 8.95 | Ferulic acid | C_10_H_10_O_4_ | M-H | 193.0514 | 178； 149；134；118 | b |
| 18 | 9.1 | Calycosin-7-glucoside | C_22_H_22_O_10_ | M-H | 446.1226 | 285；270；253；213 | a |
| 19 | 9.23 | Diethyl phthalate | C_12_H_14_O_4_ | M+Na | 223.0593 | 207；193；177；165；149 | b |
| 20 | 9.54 | Odoratin-7-O-β-D-glucoside | C_23_H_24_O_11_ | M+FA-H | 476.1322 | 475；453；315 | a |
| 21 | 9.83 | Rhamnocitin-3-O-glucoside | C_22_H_22_O_11_ | M+H | 462.1167 | 463；485；301 | a |
| 22 | 10.04 | Kaempferol | C_15_H_10_O_6_ | M-H | 285.0417 | 245；227；174 | a |
| 23 | 10.58 | Calycosin-7-O-β-D-glucopyranoside-6″-O-malonate | C_25_H_25_O_13_ | M+H | 533.1307 | 285；253；213 | a |
| 24 | 10.93 | Coumarin | C_9_H_6_O_2_ | M-H | 145.0298 | 130；103；91 | a |
| 25 | 11.05 | Isomucronulatol 7,2'-di-O-glucoside | C_29_H_38_O_15_ | M+FA-H | 671.2171 | 629；579；238 | a |
| 26 | 11.24 | nonanedioic acid | C_9_H_16_O_4_ | M-H | 187.0984 | 169；125 | b |
| 27 | 11.38 | Pratensein-7-O-glucoside | C_22_H_22_O_11_ | M+H | 463.1230 | 397；302；291；243；153 | a |
| 28 | 11.42 | 9,10-dimethoxypterocarpan-3-O-β-glucoside | C_23_H_26_O_10_ | M+FA-H | 507.1531 | 301；209 | a |
| 29 | 11.56 | Dihydroxydimethoxyisoflavones | C_16_H_12_O_5_ | M-H | 283.062 | 268；239；211 | a |
| 30 | 11.85 | Chitranone | C_22_H_14_O_6_ | M-H | 373.075 | 317；283；175 | a |
| 31 | 12.33 | Linolenic acid | C_18_H_30_O_2_ | M+H | 278.2246 | 261；95 | a |
| 32 | 12.64 | Ononin | C_22_H_22_O_9_ | M+H | 430.1275 | 269；237 | a |
| 33 | 12.83 | Linolic acid | C_18_H_32_O_2_ | M-H | 280.2402 | 263 | a,b |
| 34 | 13.65 | Mucronulatol | C_17_H_18_O_5_ | M-H | 301.0719 | 175；131 | a |
| 35 | 13.85 | Astrapterocarpan | C_23_H_26_O_10_ | M+H | 462.1524 | 327；301 | a |
| 36 | 14.05 | Octanal | C_8_H_16_O | M-H | 127.1127 | 99 | b |
| 37 | 14.47 | Calycosin | C_16_H_12_O_5_ | M+H | 284.0699 | 270；253；225 | a |
| 38 | 14.52 | Isomucronulatol | C_17_H_18_O_5_ | M+H | 303.1236 | 285；270；213；167 | a |
| 39 | 14.53 | Isomucronulatol 7-O-glucoside | C_23_H_28_O_10_ | M-H | 464.1685 | 463；283；268 | a |
| 40 | 14.81 | Formosan-7-O-β-D-glucopyranoside 6 ″-malonate | C_25_H_25_O_12_ | M+H | 517.1334 | 269；237 | a |
| 41 | 15.18 | Kumatakenin | C_17_H_14_O_6_ | M+H | 314.0804 | 269；174 | a |
| 42 | 15.42 | Medicarpin | C_16_H_14_O_4_ | M+H | 270.0832 | 238；227；173；147 | a |
| 43 | 15.73 | 9,10-dimethoxypterocarpan-3-O-β-glucoside-6-O-malonate | C_26_H_29_O_13_ | M+Na | 571.1445 | 587；549；485；301；191；167 | a |
| 44 | 15.82 | 3'-hydroxy-5'-methoxyisoflavone-7-O-glucoside | C_22_H_22_O_10_ | M+H | 446.1214 | 285；270 | a |
| 45 | 16.21 | Cinnamic acid | C_9_H_8_O_2_ | M-H | 147.0811 | 134；121；112；86 | b |
| 46 | 17.08 | Isorhamnrtin | C_16_H_12_O_7_ | M-H | 315.0519 | 293；169 | a |
| 47 | 17.34 | Coniferyl ferulate | C_20_H_20_O_6_ | M+Na | 356.1261 | 311；221；175 | b |
| 48 | 17.73 | 3-Hydroxy-9,10-Dimethoxypterocarpan | C_17_H_16_O_5_ | M+H | 300.0969 | 191；167；134 | a |
| 49 | 17.84 | Sedanolide | C_12_H_18_O_2_ | M+FA-H | 237.0568 | 180；163；149；138 | b |
| 50 | 17.98 | Isoastragaloside IV | C_41_H_68_O_14_ | M+H | 785.4637 | 584；552；457；426；352；261； | a |
| 51 | 18.16 | Astragaloside V | C_47_H_78_O_19_ | M+H | 946.5079 | 469；353；277；191 | a |
| 52 | 18.23 | （3-N-）Butylphthalide | C_12_H_14_O_2_ | M+Na | 191.1086 | 173；145 | b |
| 53 | 18.36 | Formononetin | C_16_H_12_O_4_ | M+H | 268.0748 | 254；213；198；181 | a |
| 54 | 18.48 | Astragaloside IV | C_41_H_68_O_14_ | M+Na | 807.4494 | 785；767；587；473；437；419； | a |
| 55 | 18.57 | (6αR, 11αR)-10-Hydroxy-3,9-dimethoxy-pterocarpan | C_17_H_16_O_5_ | M+Na | 300.1002 | 191；167；134 | a |
| 56 | 18.88 | Soyasaponin I | C_48_H_78_O_18_ | M-H | 942.5164 | 941；871；329 | a |
| 57 | 18.96 | Astragaloside Ⅱ | C_43_H_70_O_15_ | M+Na | 826.4696 | 809；647；629；455；437；340； | a |
| 58 | 19.02 | Senkyunolide E | C_12_H_12_O_3_ | M+H | 203.0716 | 191；187；145； | b |
| 59 | 19.36 | Senkyunolide Z | C_12_H_12_O_3_ | M-H | 203.0716 | 175；147 | b |
| 60 | 19.38 | Isoastragaloside Ⅱ | C_43_H_70_O_15_ | M+H | 826.468 | 809；647；629；455；437；143 | a |
| 61 | 19.50 | senkyunolide A | C_12_H_16_O_2_ | M+H | 193.1507 | 175；147；137；119；105；91 | b |
| 62 | 20.11 | Astragaloside Ⅰ | C_45_H_72_O_16_ | M+Na | 868.4807 | 689；671；653；455；437；217； | a |
| 63 | 20.19 | Isoastragaloside Ⅰ | C_45_H_72_O_16_ | M+H | 868.4808 | 689；671；653；455；437；419； | a |
| 64 | 20.55 | E-ligustilide | C_12_H_14_O_2_ | M+H | 190.1012 | 173；163；155；145；117 | b |
| 65 | 21.09 | Z-ligustilide | C_12_H_14_O_2_ | M+H | 190.1013 | 173；163；155；145；117 | b |
| 66 | 21.13 | Agroastragaloside Ⅲ | C_51_H_82_O_21_ | M-H | 1029.5340 | 969；469；353；277；191 | a |
| 67 | 21.18 | (3Z,3Z')6.8',7.3'－diligustilide | C_12_H_14_O_2_ | M+H | 191.1086 | 173；163；145；117；105 | b |
| 68 | 22.16 | AcetylastragalosideⅠ | C_47_H_74_O_17_ | M+H | 910.4905 | 731；713；695；437；419；143 | a |
| 69 | 22.43 | Oleanolic acid | C_30_H_48_O_3_ | M-H | 455.3523 | 293；175；117 | a |
| 70 | 22.86 | Levistolide A | C_24_H_28_O_4_ | M+H | 380.1999 | 363；338；191；173 | b |

***Note:*** *a: Astragali radix, b: Angelicae sinensis radix.*

**Supplementary Table S8.** Identification of the components in serum after oral administration of DBD

| **NO.** | **R_t_**  **(min)** | **Formula** | **Component** | **Neutral mass (Da)** | **Observedm/z** | **Mass error (mDa)** | **Ion mode** | **Fragment** | **Origin** |
| --- | --- | --- | --- | --- | --- | --- | --- | --- | --- |
| 1 | 0.44 | C_8_H_4_O_3_ | Phthalic anhydride | 148.0160 | 149.0231 | -0.2 | M+H | 122；93 | b |
| 2 | 0.76 | C_22_H_22_O_10_ | Calycosin-7-glucoside | 447.1024 | 446.1226 | 0.7 | M-H | 285；270；253；213 | a |
| 3 | 2.97 | C_9_H_6_O_2_ | Coumarin | 146.0368 | 147.0439 | -1.6 | M+H | 103 | a |
| 4 | 5.85 | C_19_H_19_N_7_O_6_ | Folic acid | 441.1397 | 440.1396 | 0.5 | M-H | 197；183 | a |
| 5 | 6.82 | C_10_H_10_O_7_S | Ferulic acid sulfate | 274.0147 | 275.0056 | -1.8 | M+H | 193；178；175；149；118 | b |
| 6 | 7.1 | C_17_H_20_N_4_O_6_ | Riboflavin | 377.1384 | 376.1456 | 0.2 | M-H | 359；282；243 | a |
| 7 | 12.17 | C_30_H_50_O_5_ | Cycloastragenol | 491.7699 | 492.7793 | 0.5 | M+H | 425；185；110 | a |
| 8 | 13.74 | C_16_H_12_O_5_ | Calycosin | 284.0685 | 285.0752 | -0.6 | M+H | 270；253；225 | a |
| 9 | 14.06 | C_23_H_26_O_10_ | 9,10-Dimethoxy-pterocarpan-3-O-β-D-glucoside | 463.1526 | 462.1576 | -2.3 | M-H | 301；209 | a |
| 10 | 14.35 | C_17_H_16_O_5_ | (6αR, 11αR)-10-Hydroxy-3,9-dimethoxy-pterocarpan | 300.0998 | 301.1068 | -0.9 | M+H | 191；167；134 | a |
| 11 | 14.5 | C_23_H_24_O_11_ | Odoratin-7-O-β-D-glucoside | 432.1435 | 431.1417 | 0.6 | M-H | 353；215 | a |
| 12 | 17.54 | C_12_H_18_O_2_ | Sedanolide | 194.1307 | 193.1274 | 0.1 | M-H | 193；171 | b |
| 13 | 18.26 | C_16_H_12_O_4_ | Formononetin | 268.0736 | 269.0811 | 0.3 | M+H | 254；213；198；181 | a |
| 14 | 18.73 | C_12_H_14_O_2_ | (Z)-Ligustilide+O | 191.1086 | 190.1081 | 0.7 | M-H | 161；135 | b |
| 15 | 19.08 | C_41_H_68_O_14_ | Astragaloside IV | 826.4715 | 849.4494 | -1.6 | M+Na | 767；587；473；455；437；419 | a |
| 16 | 19.54 | C_47_H_74_O_17_ | Acetylastragaloside Ⅰ | 910.4926 | 909.4835 | 1.7 | M-H | 731；713；695；437；419；143 | a |
| 17 | 19.67 | C_12_H_18_O_2_ | Cnidilide | 194.1307 | 195.1377 | -0.3 | M+H | 137；120 | b |
| 18 | 19.93 | C_18_H_30_O_2_ | Linolenic acid | 278.2246 | 279.2324 | 0.6 | M+H | 261；95 | a |
| 19 | 19.95 | C_18_H_32_O_2_ | Linolic acid | 280.2402 | 303.2309 | 1.5 | M-H | 265；263 | a |
| 20 | 20.9 | C_51_H_82_O_21_ | Agroastragaloside Ⅲ | 1030.5349 | 1031.5348 | -4.6 | M+H | 969；469；353；277；191 | a |

**Note:** *a: Astragali radix, b: Angelicae sinensis radix.*

**Supplementary Table S9.** Correlation analysis results of effective components and biomarkers

| **No.** | **Constituents** | **Relevant quantity** | **Source** |
| --- | --- | --- | --- |
| 1 | Phthalic anhydride | 2 | b |
| 2 | Sedanolide | 3 | b |
| 3 | Odoratin-7-O-β-D-glucoside | 3 | a |
| 4 | Folic acid | 2 | a |
| 5 | Riboflavin | 5 | a |
| 6 | Calycosin | 4 | a |
| 7 | 9,10-Dimethoxy-pterocarpan-3-O-β-D-glucoside | 3 | a |
| 8 | (6αR, 11αR)-10-Hydroxy-3,9-dimethoxy-pterocarpan | 5 | a |
| *9 | Calycosin-7-glucoside | 6 | a |
| 10 | Formononetin | 1 | a |
| *11 | Coumarin | 6 | a |
| *12 | Astragaloside IV | 7 | a |
| *13 | Acetylastragaloside Ⅰ | 6 | a |
| 14 | Cnidilide | 4 | b |
| *15 | Linolic acid | 7 | a,b |
| 16 | Linolenic acid | 2 | a |
| 17 | Agroastragaloside Ⅲ | 4 | a |
| *18 | (Z)-Ligustilide+O | 6 | b |
| *19 | Cycloastragenol | 6 | a |
| *20 | Ferulic acid sulfate | 7 | a |

**Note:** *a: Astragali radix, b: Angelicae sinensis radi*
